# The p-MYH9/USP22/HIF-1α axis promotes lenvatinib resistance and cancer stemness in hepatocellular carcinoma

**DOI:** 10.1038/s41392-024-01963-5

**Published:** 2024-09-19

**Authors:** Qiaonan Shan, Lu Yin, Qifan Zhan, Jiongjie Yu, Sheng Pan, Jianyong Zhuo, Wei Zhou, Jiaqi Bao, Lincheng Zhang, Jiachen Hong, Jianan Xiang, Qingyang Que, Kangchen Chen, Shengjun Xu, Jingrui Wang, Yangbo Zhu, Bin He, Jingbang Wu, Haiyang Xie, Shusen Zheng, Tingting Feng, Sunbin Ling, Xiao Xu

**Affiliations:** 1 Department of Hepatobiliary and Pancreatic Surgery, NHC Key Laboratory of Combined Multi-organ Transplantation, The First Affiliated Hospital, Zhejiang University School of Medicine, Hangzhou, 310058 China; 2 Department of Hepatobiliary and Pancreatic Surgery, The Center for Integrated Oncology and Precision Medicine, Affiliated Hangzhou First People’s Hospital, School of Medicine, Westlake University, Hangzhou, 310006 China; 3 Department of Hepatobiliary and Pancreatic Surgery and Minimally Invasive Surgery, Zhejiang Provincial People’s Hospital (Affiliated People’s Hospital), School of Clinical Medicine, Hangzhou Medical College, Hangzhou, 314408 China; 4 Institute of Translational Medicine, Zhejiang University, Hangzhou, 310009 China; 5 Zhejiang Cancer Hospital, Hangzhou, 310022 China

**Keywords:** Cancer stem cells, Drug development, Tumour biomarkers

## Abstract

Lenvatinib is a targeted drug used for first-line treatment of hepatocellular carcinoma (HCC). A deeper insight into the resistance mechanism of HCC against lenvatinib is urgently needed. In this study, we aimed to dissect the underlying mechanism of lenvatinib resistance (LR) and provide effective treatment strategies. We established an HCC model of acquired LR. Cell counting, migration, self-renewal ability, chemoresistance and expression of stemness genes were used to detect the stemness of HCC cells. Molecular and biochemical strategies such as RNA-sequencing, immunoprecipitation, mass spectrometry and ubiquitination assays were used to explore the underlying mechanisms. Patient-derived HCC models and HCC samples from patients were used to demonstrate clinical significance. We identified that increased cancer stemness driven by the hypoxia-inducible factor-1α (HIF-1α) pathway activation is responsible for acquired LR in HCC. Phosphorylated non-muscle myosin heavy chain 9 (MYH9) at Ser1943, p-MYH9 (Ser1943), could recruit ubiquitin-specific protease 22 (USP22) to deubiquitinate and stabilize HIF-1α in lenvatinib-resistant HCC. Clinically, p-MYH9 (Ser1943) expression was upregulated in HCC samples, which predicted poor prognosis and LR. A casein kinase-2 (CK2) inhibitor and a USP22 inhibitor effectively reversed LR in vivo and in vitro. Therefore, the p-MYH9 (Ser1943)/USP22/HIF-1α axis is critical for LR and cancer stemness. For the diagnosis and treatment of LR in HCC, p-MYH9 (Ser1943), USP22, and HIF-1α might be valuable as novel biomarkers and targets.

## Introduction

Liver cancer is one of the most prevalent malignancies and ranks third among all cancer-related causes of death.^1^ Hepatocellular carcinoma (HCC) accounts for approximately 90% of cases of primary liver cancer. Even though surveillance programs for HCC have been enhanced, patients are usually diagnosed at an intermediate or advanced stage, losing the opportunity for radical operation.^2^ Interventional therapies such as radiofrequency ablation (RFA), transarterial chemoembolization (TACE), and hepatic arterial infusion chemotherapy (HAIC) use invasive procedures to block the blood supply and deliver chemotherapy drugs directly to destroy tumor cells. Systemic treatment has transformed the way advanced HCC is managed in clinical practice, providing new opportunities to reduce tumor size and potentially enable curative surgery.^3^ Tyrosine kinase inhibitors (TKIs), such as lenvatinib, apatinib, and bevacizumab, have been shown to inhibit the angiogenesis of cancer and are widely used in HCC patients. However, consistent use will eventually result in the emergence of targeted drug resistance.

Lenvatinib is a multi-target TKI that inhibits VEGFR1-3, FGFR1-4, PDGFR-α, KIT and RET. In the REFLECT study, lenvatinib showed noninferiority to sorafenib in overall survival but demonstrated superior progression-free survival, providing another promising option for patients with HCC. It has become the second authorized first-line medication for advanced HCC after sorafenib.^4^ Previous studies have revealed that resistance to lenvatinib might be the primary limiting factor in achieving efficiency. Hyperactivation of BCL2 interacting protein3 (BNIP3) reprograms glycolysis to increase lenvatinib resistance (LR) in HCC.^5^ From a detailed analysis of heterogeneity of HCC by patient-derived tumor xenograft organoids (PDOs), a key molecule c-Jun was identified to promote stemness of HCC and LR through the β-catenin pathway. PKUF-01, a c-Jun inhibitor, has a synergistic effect with lenvatinib in LR PDOs.^6^ N6-Methyladenosine reader YTHDF1 promotes stemness and LR in PDOs and HCC cell lines by increasing *NOTCH* mRNA.^7^ Nonetheless, the mechanisms involved in LR are still unclear. It is crucial to investigate the underlying molecular mechanisms and search for potential targets to overcome LR.^8^

Cancer stem cells (CSCs) have tumorigenesis, self-renewal, metastasis, and chemotherapy resistance abilities, and are widely considered to be drivers of HCC progression.^9^ It has been reported that activation of the Notch, Wnt/β-catenin and Hippo signaling pathways contributes to HCC stemness, and targeting these pathways may restore LR.^10,11^ It is crucial to comprehend the molecular mechanisms that drive cancer stemness in HCC, in order to create targeted therapies that can effectively eliminate these aggressive cancer cells and enhance patient outcomes. Several markers such as CD90, EPCAM, CD133, OCT4 and CD44 have been found to be highly expressed in HCC and are associated with stemness.^9^ The identification and characterization of specific markers and surface proteins associated with HCC CSCs have provided opportunities for the development of novel diagnostic and therapeutic strategies. By targeting these specific markers, it may be possible to selectively eliminate CSCs and prevent tumor recurrence and metastasis.^12^ Hypoxia-inducible factor-1α (HIF-1α) and HIF-1β form the oxygen-sensitive transcription factor HIF-1, a major transcription factor that promotes the progression of a variety of tumors.^13,14^ HIF-1α participates in the development of CSCs by regulating the transcription of target genes.^15^ Under hypoxia, reduced oxygen leads to stabilization of HIF-1α. HIF-1α dimerizes with HIF-1β and translocates into the nucleus to bind to hypoxia response elements (HREs). HIF-1α is reported to prompt OCT4 and SOX2 to bind to the PROM1 promoter and increase the population of stem cells.^16^ HIF-1α directly targets the Hippo pathway effector, TAZ and regulates cancer stemness.^17^ The Notch, and Wnt/β-catenin pathways are also mediated by HIF-1α in CSCs.^18^ In addition, HIF-1α drives the transcription of the enzymes GLUT1, HK2, and LDHA, which are the key enzymes of tumor glycolysis.^19^ HIF-1α contributes to drug resistance, including chemoresistance and TKIs. MUC1 prevents HIF-1α from degradation and enhances the chemoresiatance between which EGLN2 and NF-κB may play important roles.^20^ In renal cell carcinoma, DEPDC1 activates the AKT/mTOR/HIF-1α pathway to promote glycolysis and TKI resistance.^21^ In previous studies, our group proved the importance of HIF-1α in glycolysis and cancer stemness in HCC under hypoxia.^9^ Additionally, USP22, a ubiquitin- specific protease, could deubiquitinate HIF-1α and enhance the cancer stemness and glycolysis. In solid tumors, regions with low oxygen levels are common. Overexpression of HIF-1α is found in the solid tumors, leading to changes in stemness. Mechanistically, hypoxia prevents HIF-1α from degradation by E3 ubiquitin ligase and stabilizes the protein of HIF-1α, thus displaying its function. However, the function of HIF-1α under normoxia is as important as that under hypoxia, as it can also effectively promote glycolysis and cancer stemness in cancer cells.^22–24^

In this study, we constructed HCC LR models and found that HIF-1α plays a significant role in HCC LR cells even under normoxia. An axis focused on HIF-1α was found to be responsible for promoting cancer stemness and LR in HCC. We aimed to provide insights into the mechanisms underlying LR and help identify potential targets for developing effective therapeutic strategies.

## Results

### Acquired lenvatinib-resistant HCC cells display increased cancer stemness

**Fig. 1:**
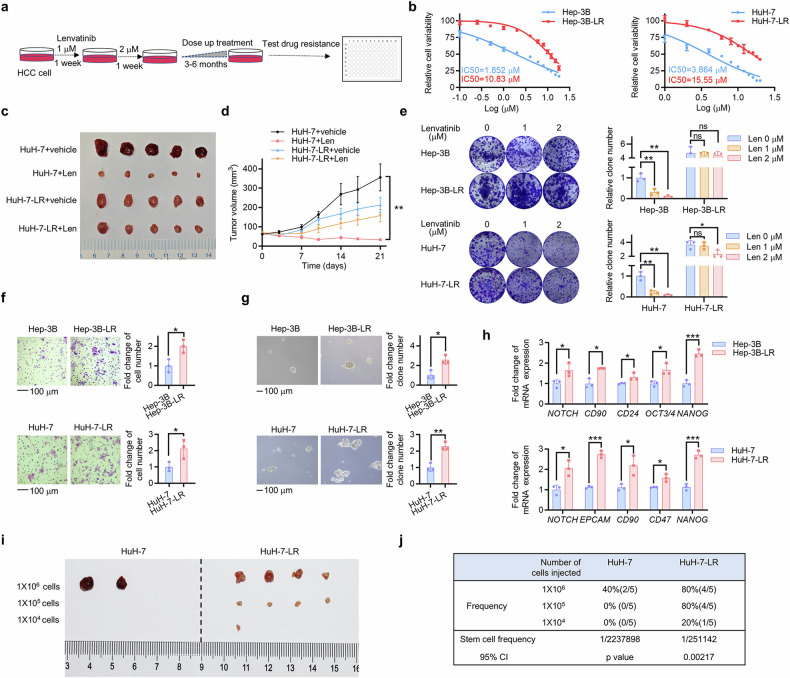
Acquired lenvatinib-resistant HCC cells display increased cancer stemness. **a** HCC cells were subjected to escalating doses of lenvatinib over a period of 3–6 months in order to develop lenvatinib-resistant cell models. **b** The IC50 value of Hep-3B, Hep-3B-LR, HuH-7, and HuH-7-LR cells was determined via CCK-8 assay. Each point on the dose-response curves consisted of five replicates. **c** A vehicle or a Len treatment was administered to nude mice xenografted with HuH-7 cell (lenvatinib, 30 mg/kg) for 3 weeks (*n* = 5 per group). The isolated tumors were photographed after the mice were sacrificed. **d** Tumor volume of each group was measured twice a week. **e** Hep-3B, Hep-3B-LR, HuH-7, and HuH-7-LR cells were treated with the indicated concentrations of lenvatinib. Then, the remaining cells were stained after 2 weeks with crystal violet staining. The colony formations were photographed and counted. Histograms were used to statistically analyze changes. **f** The migration ability of Hep-3B, Hep-3B-LR, HuH-7, and HuH-7-LR cells was evaluated by transwell assay after incubation for 24 hours. **g** The sphere formation assay was used to evaluate the in vitro self-renewal ability. **h** The qRT-PCR assay was employed to measure the mRNA levels of stemness markers. **i** Limiting dilution xenograft formation of HuH-7 and HuH-7-LR cells in NOD/SCID mice (*n* = 5 per group). The isolated tumors were photographed, and the stem cell frequency (**j**) was calculated. Extreme limiting dilution analysis (ELDA) was used for the limiting dilution assay. The data from cell functional assays and RT-qPCR analysis were presented as mean ± SD of three individual experiments, and the data from animal experiments were presented as mean ± SEM. The Student’s *t* test was used for comparisons. *p < 0.05, **p < 0.01, ***p < 0.001. Scale bar: 100 μm. LR lenvatinib resistant, CCK-8 Cell Counting Kit-8, ns nonsignificant

To establish HCC LR cell models, we exposed Hep-3B, HuH-7 and SNU-387 cells to increasing concentrations of lenvatinib (starting from 2 μM) for 3–6 months (Fig. 1a) and named them Hep-3B-LR, HuH-7-LR and SNU-387-LR. Compared with the parent strains, the LR strains acquired elevated IC50 values from 1.85 μM to 10.83 μM, 3.86 μM to 15.55 μM and 11.89 μM to 19.36 μM (Fig. 1b; Supplementary Fig. 1a). Nude mouse model bearing HuH-7 cell and HuH-7-LR cell xenografts were used to elucidate the effect of LR in vivo. HuH-7 cell xenografts showed a marked reduction in tumor volume following the administration of lenvatinib, while HuH-7-LR cell xenografts were decreased slightly (Fig. 1c, d). To avoid the side effects of lenvatinib, the weights of the mice were obtained, and no significant variation was found (Supplementary Fig. 1b). The colony formation assay further confirmed the LR phenotype (Fig. 1e; Supplementary Fig. 1c). Considering drug resistance as a crucial element of cancer stemness, we explored changes in stemness properties in HCC LR cells. Compared with the parent strains, the LR strains displayed significantly promoted migration ability (Fig. 1f; Supplementary Fig. 1d), formation of more and larger hepatospheres (Fig. 1g; Supplementary Fig. 1e) and upregulated mRNA expression of CSC markers (Fig. 1h; Supplementary Fig. 1f). We also examined the tumorigenicity of HuH-7 and HuH-7-LR cells in vivo (Fig. 1i). The estimated CI for the frequency of CSCs in HuH-7-LR cells was 1/251142, while that in HuH-7 cells was 1/2237898 (Fig. 1j). These results suggested that the LR strains displayed increased cancer stemness.

### HIF-1α pathway activation is responsible for acquired LR and increased cancer stemness in HCC

**Fig. 2:**
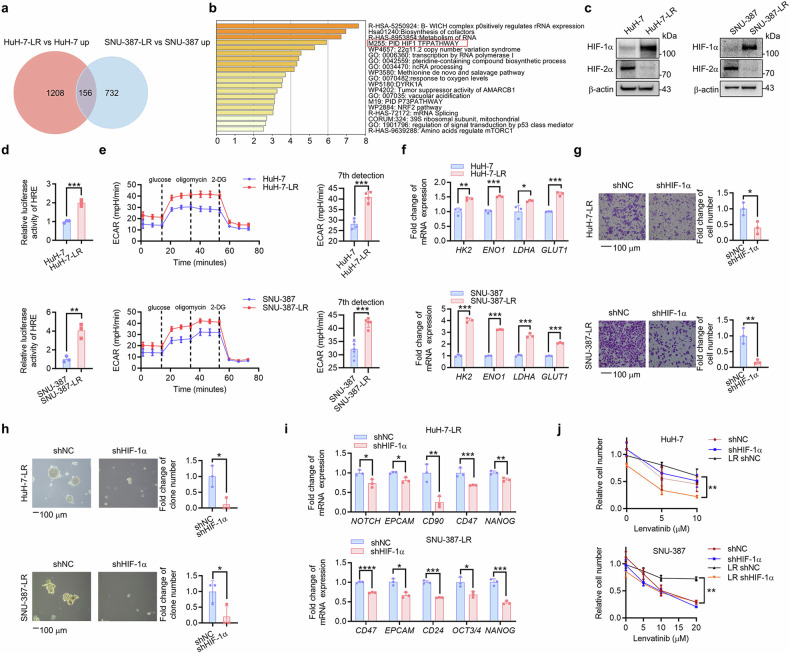
HIF-1α pathway activation is responsible for acquired LR and increased cancer stemness in HCC. **a** Top 156 upregulated DEGs of HuH-7-LR versus HuH-7 and SNU-387-LR versus SNU-387 were extracted from RNA-seq data. **b** Upon KEGG analysis show, it was revealed that the 156 upregulated DEGs were involved in associated with the HIF-1α pathway. **c** The expression of HIF-1α and HIF-2α was detected in WT and HCC LR cells via western blot analysis. **d** Changes in relative luciferase activity in WT and HCC LR cells were determined by luciferase reporter assay. **e** The ECAR of HCC LR cells and WT cells was identified using the Seahorse system. The means and SDs are represented on the column graphs. **f** qRT-PCR was used to measure the expression of glycolysis driver genes in WT and HCC LR cells. The effects of HIF-1α knockdown (shHIF-1α) or not (shNC) on HuH-7-LR and SNU-387-LR cell stemness were shown according to migration (**g**), in vitro self-renewal (**h**), and mRNA expression of stemness markers (**i**). **j** The WT and HCC LR cells with or without HIF-1α knockdown (shHIF-1α or shNC) were exposed to the indicated concentrations of lenvatinib for 48 hours. Trypan blue staining-based cell counting was used to detect lenvatinib sensitivity. The data were presented as mean ± SD of three individual experiments. The Student’s *t* test was used for comparisons. *p < 0.05, **p < 0.01, ***p < 0.001, ****p < 0.0001. Scale bar: 100 μm. DEGs differentially expressed genes, KEGG Kyoto Encyclopedia of Genes and Genomes, WT wild type, LR lenvatinib resistant, HIF-1α hypoxia-inducible factor-1α, HIF-2α hypoxia-inducible factor-2α, HCC hepatocellular carcinoma, ECAR extracellular acidification rate

To investigate the molecular profile of the acquired HCC LR cells, we performed RNA sequencing and found that the HIF-1-centered transcriptional pathway was significantly activated (Fig. 2a, b). As the main subunit of HIFs, HIF-1α protein was found to be increased in HCC LR cells, while the HIF-2α protein had unstable changes (Fig. 2c; Supplementary Fig. 2a), which further supported the RNA-sequencing results. HIF-1α transcriptional activity was also increased in HCC LR cells (Fig. 2d; Supplementary Fig. 2b). The HIF-1α pathway is known to play a critical role in both glycolysis and cancer stemness. Thus, we tested the glycolysis capacity by a Seahorse XF Analyzer and found a higher extracellular acidification rate (ECAR) in HuH-7-LR and SNU-387-LR cells (Fig. 2e). In LR cells, the mRNA levels of glycolysis-related genes were elevated (Fig. 2f; Supplementary Fig. 2c). Moreover, knockdown of HIF-1α effectively decreased the migration ability (Fig. 2g), in vitro self-renewal ability (Fig. 2h) and the mRNA expression of CSC markers (Fig. 2i; Supplementary Fig. 2d). To identify the role of HIF-1α in LR, parent cells and LR cells were exposed to lenvatinib with or without HIF-1α knockdown. The results showed that HIF-1α knockdown could markedly increase the sensitivity of HCC LR cells to lenvatinib (Fig. 2j), but there was no significant difference in parent cells. In summary, these data revealed that activation of HIF-1α pathway promoted cancer stemness and LR in HCC.

### MYH9 promotes LR and cancer stemness in HCC through stabilizing HIF-1α

**Fig. 3:**
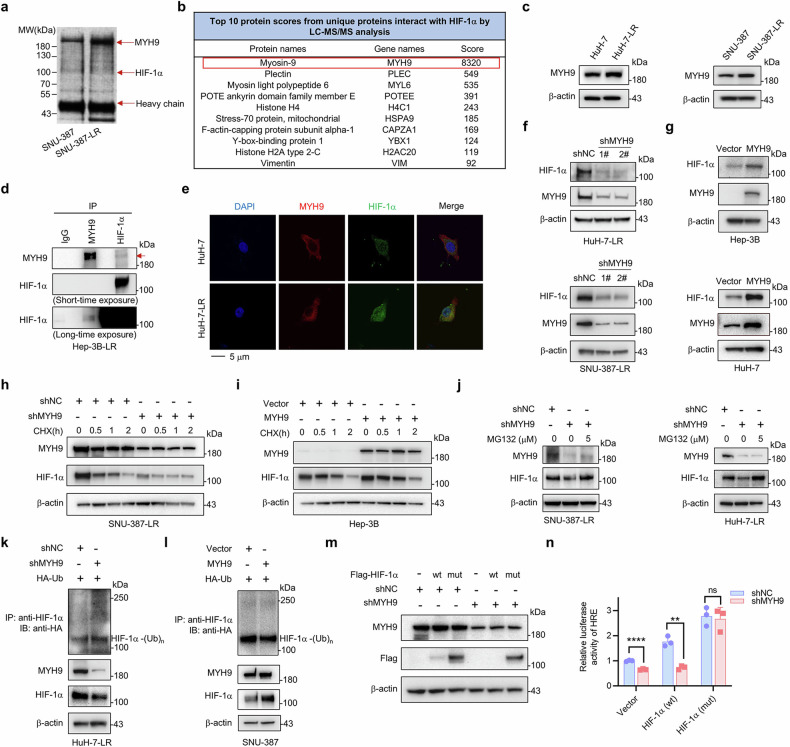
Identifying MYH9 as a HIF-1α-interacting protein and the effect of MYH9 on HIF-1α protein stabilization and ubiquitination. **a** Immunoprecipitated proteins of SNU-387 and SNU-387-LR cells were separated by SDS-PAGE and visualized by silver staining. **b** The top 10 protein scores from unique proteins that interact with HIF-1α were identified by LC-MS/MS analysis. **c** Western blot was conducted to assess the MYH9 expression in WT and HCC LR cells. **d** The interaction between endogenous MYH9 and HIF-1α was tested in Hep-3B-LR cells. IgG was used as a control. The MYH9 protein is indicated by the red arrow. **e** Colocalization of HIF-1α (green) with MYH9 (red) was observed by confocal microscopy in HuH-7 and HuH-7-LR cells. The nuclei were stained with DAPI. **f** Western blot analysis was used to assess the protein expression of MYH9 and HIF-1α following MYH9 knockdown (shMYH9) or shNC in HCC LR cells. **g** Western blot analysis was used to assess the protein expression of MYH9 and HIF-1α in WT HCC cells with MYH9 overexpression (MYH9) or vector. SNU-387-LR cells were treated with shMYH9 or shNC. **h** While Hep-3B cells were treated with MYH9 or vector (**i**). Both cell lines were then exposed to 10 μg/mL CHX for the designed time. The protein levels of HIF-1α and MYH9 were determined via western blot analysis. **j** SNU-387-LR and HuH-7-LR cells were treated with shMYH9 or shNC, and then exposed to MG132 (5 μM) for 2 hours. Western blot analysis was performed to assess the protein levels of HIF-1α and MYH9. **k** HuH-7-LR cells were cotransfected with shMYH9 or shNC and Ub (HA-tag) plasmids. **l** SNU-387 cells were cotransfected with MYH9 overexpression or vector and HA-Ub plasmids. Anti-HIF-1α antibody was used to isolate endogenous HIF-1α and anti-HA antibody was used to detect bound Ub. Exogenous MYH9 and HIF-1α expression in whole cell lysates was detected. **m** HuH-7-LR cells with shMYH9 or shNC were stably overexpressed with HIF-1α (wt) or mutated HIF-1α (P402A, P564A), HIF-1α (mut), which were with Flag-tag. Flag and MYH9 protein were determined by western blot, while the relative luciferase activity of HRE was analyzed (**n**). The data were presented as mean ± SD of three individual experiments. The Student’s *t* test was used for comparisons. **p < 0.01, ****p < 0.0001. Scale bar: 5 μm. IP immunoprecipitation, MYH9 non-muscle myosin heavy chain 9, WT wild type, LR lenvatinib resistant, HIF-1α hypoxia-inducible factor-1α, HCC hepatocellular carcinoma, CHX cycloheximide, HRE hypoxia-responsive element, ns nonsignificant

To further determine the mechanism underlying the increased HIF-1α protein in HCC LR cells, we conducted immunoprecipitation with an anti-HIF-1α antibody on the whole cell lysate from SNU-387 and SNU-387-LR cells. After analyzing the results of silver staining and mass spectrometry sequencing, MYH9 was found to be a potential potent HIF-1α-interacting protein (Fig. 3a, b) in HCC LR cells. MYH9 was also increased in HCC LR cells (Fig. 3c; Supplementary Fig. 3a). The exogenous and endogenous interaction of HIF-1α and MYH9 was also confirmed in HEK-293T and HCC LR cells, respectively (Fig. 3d; Supplementary Fig. 3b). Immunofluorescence analysis revealed that the protein expression of HIF-1α and MYH9 was elevated in HuH-7-LR cells compared to that in HuH-7 cells and their colocalization was in cytoplasm (Fig. 3e). Given that HIF-1α could be stabilized by its interacting proteins, we speculated that MYH9 could bind to and stabilize HIF-1α. We demonstrated that HIF-1α was notably decreased in MYH9 knockdown HCC LR cells (Fig. 3f; Supplementary Fig. 3c). In parent HCC cells, overexpression of MYH9 increased HIF-1α protein expression (Fig. 3g; Supplementary Fig. 3d). But mRNA expression changes of HIF-1α were unstable with MYH9 knockdown in LR cells or MYH9 overexpression in HCC cells (Supplementary Fig. 3e, f). Moreover, knockdown of endogenous MYH9 facilitated HIF-1α protein degradation and inhibited its transcriptional activity in HCC LR cells (Fig. 3h; Supplementary Fig. 3i). Conversely, overexpression of MYH9 significantly delayed HIF-1α protein degradation and promoted its transcriptional activity in parent cells (Fig. 3i; Supplementary Fig. 3g, j). The above results indicated that MYH9 could interact and stabilize the HIF-1α protein.

The proteasome-specific inhibitor MG132 rescued the HIF-1α protein from degradation in MYH9 knockdown cells (Fig. 3j; Supplementary Fig. 3h). Furthermore, MYH9 knockdown significantly enhanced polyubiquitination-induced HIF-1α degradation (Fig. 3k), while MYH9 overexpression significantly inhibited ubiquitin conjugation to HIF-1α (Fig. 3l). According to a previous study,^9^ we developed a stable HIF-1α mutant that is oxygen-independent by replacing two prolines with alanine in the oxygen-dependent degradation domain (ODDD) (P402 and P564). Mutated HIF-1α (P402 and P564) significantly rescued the protein level of HIF-1α in MYH9 knockdown cells (Fig. 3m). MYH9 knockdown had no influence on the transcriptional activity of the mutated HIF-1α (Fig. 3n). These results suggested that MYH9 stabilized the HIF-1α protein by preventing its ubiquitination and subsequent proteasomal degradation.

**Fig. 4:**
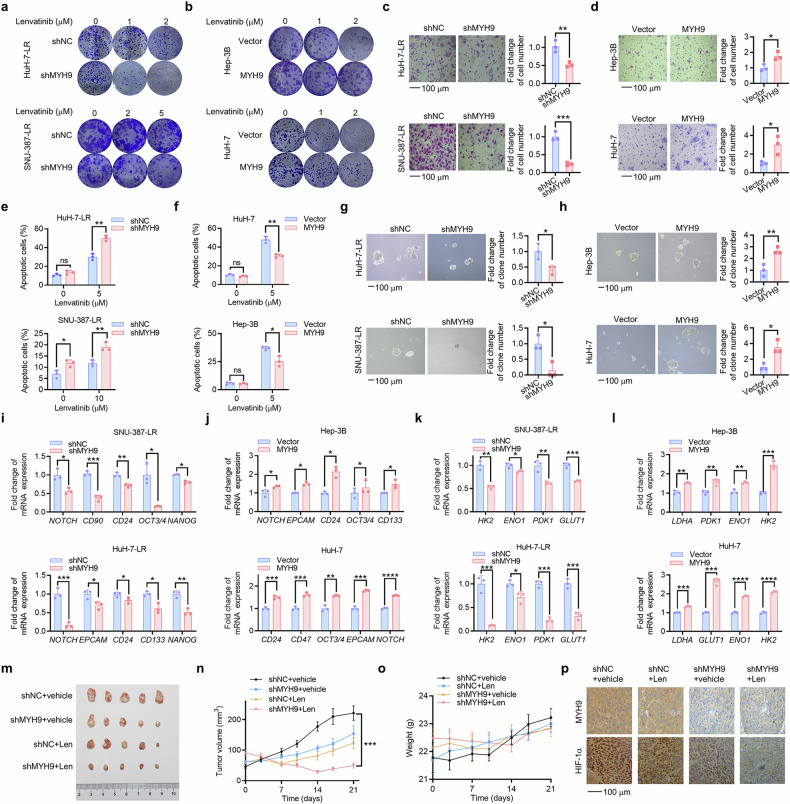
MYH9 promotes LR and cancer stemness in HCC. HCC LR cells with MYH9 knockdown (shMYH9) or shNC. **a** and WT HCC cells with MYH9 overexpression or vector (**b**) were cultured with the indicated lenvatinib concentrations. Following 14 days, crystal violet staining was used to determine the remaining cells. The influence of shNC and shMYH9 on HCC LR cell stemness was demonstrated through assessments of migration (**c**), flow cytometry (**e**), in vitro self-renewal (**g**) and the mRNA expression of stemness markers (**i**) and glycolysis driver genes (**k**). The influence of vector and MYH9 overexpression on WT HCC cell stemness was demonstrated through assessments of migration (**d**), flow cytometry (**f**), in vitro self-renewal (**h**) and the mRNA expression of stemness markers (**j**) and glycolysis driver genes (**l**). **m** HuH-7-LR cells with shMYH9 or shNC xenografted nude mice were treated with vehicle or Len (lenvatinib, 30 mg/kg) for 3 weeks after the tumor reached an average size of 50–100 mm^3^ (*n* = 5 per group). After 21 days, the mice were sacrificed. The isolated tumors were photographed. Tumor volume (**n**) and mouse weight of each group (**o**) were recorded every other week. **p** Immunohistochemistry was used to assess the expression of MYH9 and HIF-1α in the xenografts post-treatment. The data of cell functional assays and RT-qPCR analysis were presented as mean ± SD of three individual experiments, and the data of animal experiments were presented as mean ± SEM. The Student’s *t*-test was used for comparisons. *p < 0.05, **p < 0.01, ***p < 0.001, ****p < 0.0001. Scale bar: 100 μm., non-muscle myosin heavy chain 9; WT wild type, LR lenvatinib resistant, HIF-1α hypoxia-inducible factor-1α, HCC hepatocellular carcinoma, ns nonsignificant

To ascertain the involvement of MYH9 in LR and cancer stemness of HCC cells, we further conducted a series of functional experiments. MYH9 knockdown effectively suppressed colony formation under lenvatinib treatment (Fig. 4a; Supplementary Fig. 4a) and migration (Fig. 4c; Supplementary Fig. 4c) and promoted lenvatinib-induced apoptosis (Fig. 4e) in HCC LR cells. In addition, MYH9 knockdown resulted in reduced hepatosphere formation (Fig. 4g; Supplementary Fig. 4e) and decreased mRNA expression of CSC markers (Fig. 4i; Supplementary Fig. 4g) and glycolysis-related genes (Fig. 4k; Supplementary Fig. 4i) in HCC LR cells. Conversely, MYH9 overexpression significantly promoted colony formation (Fig. 4b; Supplementary Fig. 4b) and migration (Fig. 4d; Supplementary Fig. 4d) and suppressed lenvatinib-induced apoptosis (Fig. 4f) in HCC cells. MYH9 overexpression also resulted in the opposite trend in hepatosphere formation (Fig. 4h; Supplementary Fig. 4f), expression of CSC mRNA expression of CSC markers (Fig. 4j; Supplementary Fig. 4h) and glycolysis-related genes (Fig. 4l; Supplementary Fig. 4j) in HCC cells compared to HCC LR cells. Importantly, MYH9 knockdown significantly sensitized HCC LR cells to lenvatinib in vivo (Fig. 4m, n). There was no notable variance in the body weights of mice (Fig. 4o). The expression of MYH9, HIF-1α and the proliferation marker Ki67 was suppressed by the combination of MYH9 shRNA and lenvatinib (Fig. 4p; Supplementary Fig. 4k). In summary, we demonstrated that MYH9 could stabilize HIF-1α and promote LR, glycolysis and cancer stemness in HCC.

### Phosphorylated MYH9 (Ser1943) interacts with HIF-1α

**Fig. 5:**
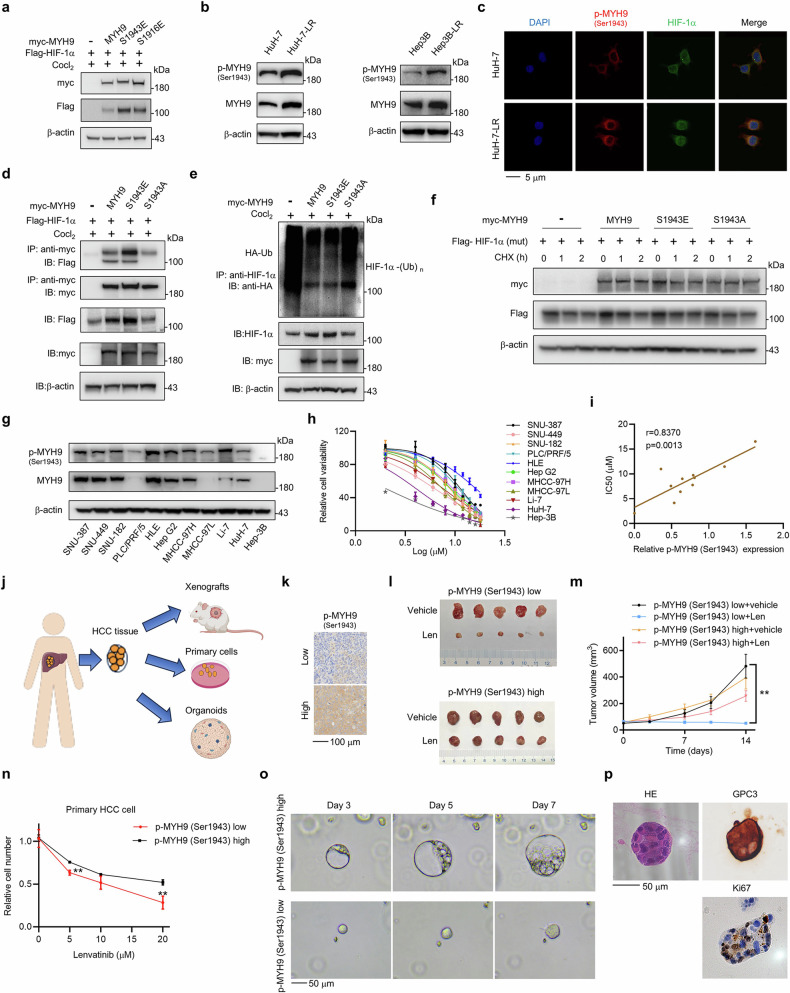
Phosphorylated MYH9 at Ser1943 interacts with HIF-1α and promotes LR and stemness in HCC. **a** HIF-1α (Flag) and MYH9 (myc) plasmids or phosphorylation mimics at S1943 (S1943E) and S1916 (S1916E) were transfected into HEK-293T cells. Anti-myc antibody was used to detect MYH9, and anti-Flag antibody represented HIF-1α. **b** The expression of p-MYH9 (Ser1943) in WT and LR HCC cells was detected using western blotting. **c** Colocalization of HIF-1α (green) with p-MYH9 (Ser1943) (red) was observed by confocal microscopy in HuH-7 and HuH-7-LR cells. The nuclei were stained with DAPI. Scale bar: 5 μm. **d** HIF-1α interactions with MYH9, MYH9-S1943E and its dephosphorylation mimic (MYH9-S1943A) were tested. Vector (–), MYH9 (wt) and its mutants (myc-tag) were transfected into HEK-293T cells respectively along with HIF-1α plasmid (Flag-tag). The immunoprecipitation assay was conducted to isolate myc-tagged vector and MYH9 protein, and anti-Flag antibody was used to detect bound HIF-1α protein. The expression of Flag-HIF-1α, myc-MYH9 and β-actin in whole cell lysates was verified. **e** MYH9, MYH9-S1943E and MYH9-S1943A (myc-tag) were transfected into HEK-293T cells respectively along with Ub plasmid (HA-tag). The immunoprecipitation assay was conducted to isolate endogenous HIF-1α and anti-HA antibody was used to detected bound Ub. The expression levels of HIF-1α and my-tagged MYH9 in whole cell lysates was verified. **f** Vector (–), MYH9 (wt) and its mutants (myc-tag) were transfected into HEK-293T cells respectively along with mutated HIF-1α (P402A, P564A) (HIF-1α (mut)) plasmids (Flag-tag). 10 μg/mL CHX was added at the designed time. Western blot was used to assess the expression of HIF-1α and MYH9. **g** The expression of p-MYH9(Ser1943) and MYH9 in multiple HCC cell lines was analyzed by western blot. **h** The IC50 of multiple HCC cell lines was measured by a CCK-8 assay to determine cell viability. Each point on the dose-response curves represents five technical replicates. **i** The correlation between IC50 and the relative p-MYH9 (Ser1943) protein expression was examined. **j** Schematic of the construction of PDXs, primary cells and PDOs models from human HCC tissues. Created with BioRender.com. **k** Selection of patients with high and low expression of p-MYH9 (Ser1943) were detected by IHC. Scale bar: 100 μm. **l** NOD/SCID mice bearing PDX with high and low expression of p-MYH9 (Ser1943) were treated with vehicle or lenvatinib for 2 weeks (*n* = 5 per group). The isolated tumors were photographed. **m** Tumor volume of each group was measured twice a week. **n** Primary cells with high or low expression of p-MYH9 (Ser1943) were treated with lenvatinib under different concentrations for 48 hours in vitro. Alive cell numbers of each group were counted. Relative cell number was calculated. **o** The relative representative bright-field microscopy images of PDO with high or low expression of p-MYH9 (Ser1943) were photographed on Day 3, 5 and 7. **p** HE staining of PDO and IHC staining of GPC3 and Ki67 in PDO with high expression of p-MYH9 (Ser1943) were detected. Scale bar: 50 μm. The relevance analysis was conducted using Pearson’s correlation. The data of cell functional assays were presented as mean ± SD of three individual experiments, and the data of animal experiment were presented as mean ± SEM. The Student’s *t* test was used for comparisons. **p < 0.01. MYH9 non-muscle myosin heavy chain 9, WT wild type, LR lenvatinib resistant, HIF-1α hypoxia-inducible factor-1α, HCC hepatocellular carcinoma, CHX cycloheximide, CCK-8 Cell Counting Kit-8, IP immunoprecipitation, PDX patient-derived xenograft PDO patient-derived organoid

Phosphorylation is a common modification of MYH9, and Ser1916 and Ser1943 are the two most reported phosphorylation sites.^25^ We generated S1916E and S1943E mutants (in which serine was substituted with glutamic acid to mimic the phosphorylated form) in constructs expressing myc-tagged MYH9. In the regulation of HIF-1α, phosphorylated MYH9 at Ser1943 had a more pronounced effect on the upregulation of HIF-1α protein than phosphorylated MYH9 at Ser1916 did (Fig. 5a). p-MYH9 (Ser1943) was also increased in HCC LR cells (Fig. 5b; Supplementary Fig. 5a). To investigate the interaction between p-MYH9 (Ser1943) and HIF-1α protein, we performed immunofluorescence staining and found that p-MYH9 (Ser1943) colocalized with HIF-1α in the cytoplasm (Fig. 5c). The protein expression data indicated that p-MYH9 (Ser1943) was primarily localized in the cytoplasm, while HIF-1α was present in both the nucleus and cytoplasm of HCC LR cells (Supplementary Fig. 5b). Additionally, we generated an S1943A mutant (in which serine was substituted with alanine to mimic the unphosphorylated form) in a construct expressing myc-tagged MYH9. The MYH9-S1943E mutant, had a more potent capability to bind HIF-1α than the MYH9-S1943A mutant (Fig. 5d). Moreover, MYH9-S1943E had a potent deubiquitination effect on the HIF-1α protein, while MYH9-S1943A did not (Fig. 5e). Furthermore, the wild type MYH9 and MYH9-S1943E did not affect the degradation of mutated HIF-1α (P402, P564) (Fig. 5f). These results indicated that p-MYH9 (Ser1943) dominates the deubiquitination regulation of the HIF-1α protein.

### Targeting p-MYH9 (Ser1943) reverses LR and suppresses cancer stemness in HCC

Since MYH9 stabilized HIF-1α and promoted LR and p-MYH9 (Ser1943) specifically interacted with HIF-1α, we wondered whether p-MYH9 (Ser1943) could be an effective target for reversing LR and suppressing cancer stemness. We measured the expression level of p-MYH9 (Ser1943) in 11 liver cancer cell lines (Fig. 5g), and calculated the IC50 values of the HCC cell lines for lenvatinib by CCK-8 detection (Fig. 5h). A strong positive correlation (*r* = 0.837) was observed between p-MYH9 (Ser1943) expression and the IC50 values of the HCC cell lines for lenvatinib (Fig. 5i), revealing that p-MYH9 (Ser1943) may play an important role in driving HCC primary resistance to lenvatinib. Moreover, we constructed patient-derived HCC models, including patient-derived tumor xenografts (PDX), primary cells and PDO from human HCC tissues (Fig. 5j). According to the level of p-MYH9 (Ser1943) expression (Fig. 5k), PDX models with low p-MYH9 (Ser1943) expression demonstrated significantly greater sensitivity to lenvatinib compared to PDX models with high p-MYH9 (Ser1943) expression (Fig. 5l, m; Supplementary Fig. 5c). The primary HCC cell models displayed similar results (Fig. 5n). Since organoids are an excellent model for assessing stemness, we also conducted experiments with PDO models. Our findings revealed that PDO models exhibiting higher levels of p-MYH9 (Ser1943) expression demonstrated faster expansion compared to PDO models with lower p-MYH9 (Ser1943) expression (Fig. 5o, p).

**Fig. 6:**
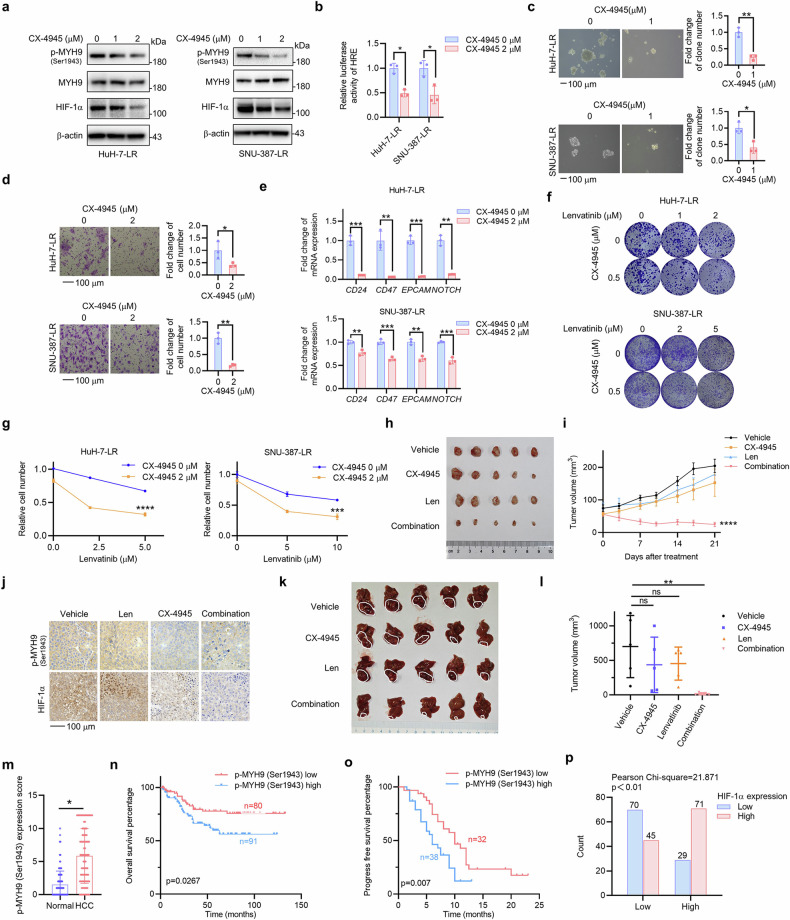
p-MYH9 (Ser1943) is a therapeutic target for stemness and LR, and predicts poor prognosis and LR in patients with HCC. **a** HuH-7-LR and SNU-387-LR cells were exposed to the indicated concentrations of CX-4945. Western blot was used to assess expression of p-MYH9 (Ser1943), MYH9 and HIF-1α (**b**) The effect of CX-4945 treatment on the relative luciferase activity of HRE in HCC LR cells was also investigated. The influence of CX-4945 on HCC LR cell stemness was used to assess in vitro self-renewal (**c**), migration (**d**), the mRNA expression of stemness markers (**e**) and colony formation (**f**). **g** HuH-7-LR and SNU-387-LR cells were treated with DMSO or 2 μM CX-4945 combined with the indicated concentration of lenvatinib. Differences in sensitivity to lenvatinib and CX-4945 were observed through cell count using trypan blue staining. **h** HuH-7-LR cell xenografted nude mice were administrated with vehicle, Len (lenvatinib, 30 mg/kg), CX-4945 (20 mg/kg) and their combination daily for 3 weeks after the tumor volume reached an average size of 50–100 mm^3^ (*n* = 5 per group). Then, the mice were sacrificed and the isolated tumors were photographed. **i** Tumor volume of each group was measured twice a week. **j** IHC was used to detect the expression of HIF-1α and p-MYH9 (Ser1943) in the xenografts following treatment. **k** HuH-7-LR cell was injected into the sub-capsular space of right liver lobe nude mice to establish the orthotopic tumor model. After 1 week, mice were treated with vehicle, Len (lenvatinib, 30 mg/kg), CX-4945 (20 mg/kg) and their combination daily for 3 weeks (*n* = 5 per group). Then, the mice were sacrificed and the isolated livers were photographed. **l** Tumor volume was calculated. **m** p-MYH9 (Ser1943) expression was detected by IHC in normal adjacent to tumor tissue (NAT) and HCC samples from 171 patients. Based on IHC staining of p-MYH9 (Ser1943), patients were categorized into two groups, and subsequent overall survival curves (**n**) were plotted. **o** Patients with HCC treated with lenvatinib were separated into two groups depending on their p-MYH9 (Ser1943) protein expression, and subsequent progression-free survival curves were plotted. **p** Positive correlation between p-MYH9 (Ser1943) and HIF-1α IHC results in 215 HCC samples. The data of cell functional assays, Luciferase reporter assay and RT-qPCR analysis were presented as mean ± SD of three individual experiments, and the data of animal experiments were presented as mean ± SEM. The Student’s *t*-test was used for comparisons. *p < 0.05, **p < 0.01, ***p < 0.001, ****p < 0.0001. Scale bar: 100 μm. MYH9 non-muscle myosin heavy chain 9, LR lenvatinib resistance, HIF-1α hypoxia-inducible factor-1α, HRE hypoxia-responsive element, HCC hepatocellular carcinoma, IHC immunohistochemistry, DMSO dimethyl sulfoxide, ns nonsignificant

Due to the lack of specific inhibitors of p-MYH9 (Ser1943), we screened several inhibitors reported by previous studies^26^ (Supplementary Fig. 6a). Compared to other inhibitors, the casein kinase-2 (CK2) inhibitor CX-4945 effectively inhibited the expression of p-MYH9 (Ser1943) and HIF-1α (Fig. 6a) and the transcriptional activity of HIF-1α (Fig. 6b). Functional assays showed that CX-4945 suppressed hepatosphere formation (Fig. 6c), migration (Fig. 6d) and mRNA expression of CSC markers (Fig. 6e) in HCC LR cells. CX-4945 combined with lenvatinib had a synergistic effect on suppressing colony formation and proliferation in HuH-7-LR, SNU-387-LR cells and primary HCC cells (Fig. 6f, g; Supplementary Fig. 6b, c). In vivo assays further demonstrated that CX-4945 could sensitize HCC LR cells to lenvatinib and inhibit the growth of xenograft tumors with acceptable adverse effect (Fig. 6h, i; Supplementary Fig. 6d). Immunohistochemical analysis of the xenograft tumors showed that the expression of p-MYH9 (Ser1943) and HIF-1α was significantly decreased in the CX-4945 and combination groups (Fig. 6j). Ki67 was suppressed by the combination of CX-4945 and lenvatinib (Supplementary Fig. 6e). Results from orthotopic HCC models also showed similar trends (Fig. 6k, l; Supplementary Fig. 6f). These results suggested that CX-4945 could inhibit the cancer stemness of LR cells in vitro and in vivo, which further proved that p-MYH9 (Ser1943)-mediated HIF-1α stability regulated the cancer stemness of HCC LR cells.

### p-MYH9 (Ser1943) is upregulated in HCC and predicts poor prognosis and LR in patients with HCC

To verify the clinical significance of p-MYH9 (Ser1943), we measured its expression in 171 HCC and normal adjacent tumor tissues by IHC and found that the p-MYH9 (Ser1943) level was significantly upregulated in HCC, which indicates that p-MYH9 (Ser1943) is a potential therapeutic target in HCC (Fig. 6m; Supplementary Fig. 6g). Survival analysis showed that higher levels of p-MYH9 (Ser1943) expression were correlated with worse patient outcomes in terms of both overall survival and recurrence-free survival (Fig. 6n; Supplementary Fig. 6h). In 70 HCC patients taking lenvatinib, progression-free survival was longer in the low p-MYH9 (Ser1943) group (Fig. 6o). A univariate Cox proportional hazard analysis revealed that poor differentiation, AFP > 400 ng/mL, microvascular invasion, AJCC stages III-IV and high expression of p-MYH9 (Ser1943) were positively related to shorter overall survival time of HCC patients. A multivariate Cox proportional hazard analysis showed that p-MYH9 (Ser1943) expression and microvascular invasion served as independent prognostic factors for HCC patients (Supplementary Table 6). Similarly, high expression of p-MYH9 (Ser1943) was also an independent prognostic factor for HCC patients taking lenvatinib (Supplementary Table 7). Moreover, the protein expression of p-MYH9 (Ser1943) and HIF-1α was assessed in 215 HCC samples. The results showed a strong positive correlation between p-MYH9 (Ser1943) and HIF-1α (Fig. 6p; Supplementary Fig. 6i), further confirming the relationship between the two proteins. Thus, clinically, high p-MYH9 (Ser1943) expression in HCC could predict poor prognosis and LR.

### p-MYH9 (Ser1943) recruits USP22 to deubiquitinate HIF-1α and promote LR

**Fig. 7:**
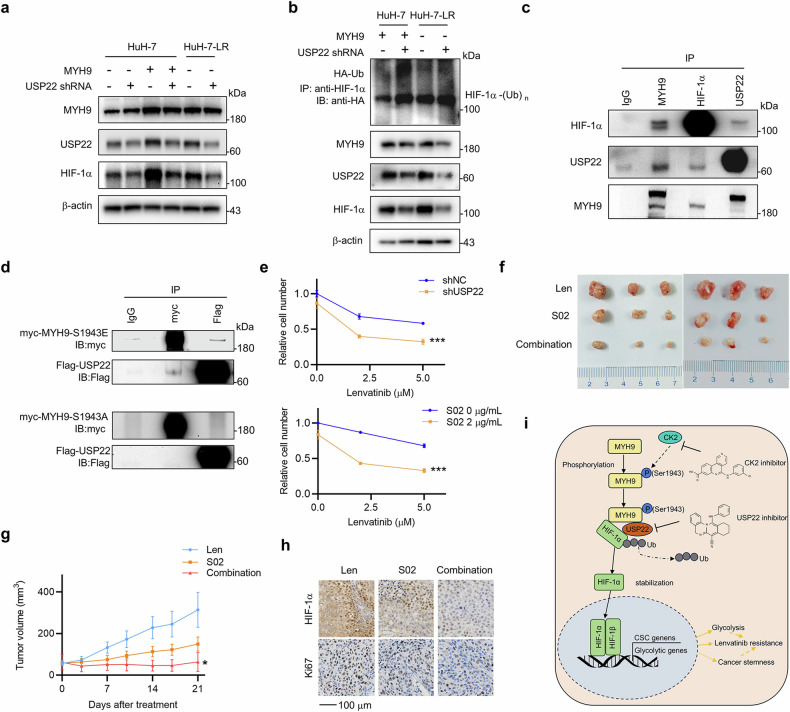
p-MYH9 (Ser1943) recruits USP22 to deubiquitinate HIF-1α and promote LR. **a** HuH-7 cells with vector and MYH9 overexpression and HuH-7-LR cells, accompanied by USP22 knockdown (shUSP22) or not, the altered protein expression of MYH9, HIF-1α and USP22 was detected through western blot. **b** Both the shUSP22 and Ub (HA-tagged) plasmids were transfected into HuH-7 cells with MYH9 overexpression and HuH-7-LR cells. Anti-HIF-1α antibody was used to isolated the HIF-1α protein and anti-HA antibody was used to detected bound Ub. MYH9, HIF-1α and USP22 protein expression levels were determined in whole cell lysates. **c** The interaction of endogenous MYH9, HIF-1α and USP22 was detected in HuH-7-LR cells. IgG was used as a control. **d** USP22 (Flag-tag) plasmid was transfected with MYH9-S1943E (myc-tag) or MYH9-S1943A (myc-tag) plasmids in HEK-293T cells. The interaction of myc protein and Flag protein was detected. IgG was used as a control. **e** HuH-7-LR cells were transfected with shNC or shUSP22, and cells were treated with DMSO or 2 μg/mL USP22 inhibitor, S02 combined with the indicated concentration of lenvatinib. Variations in lenvatinib and USP22 inhibitor sensitivity were detected by trypan blue staining-based cell count. The data were presented as mean ± SD of three individual experiments. **f** HuH-7-LR cell xenografted nude mice were fed lenvatinib (30 mg/kg), S02 (10 mg/kg) and their combination daily for three weeks after the tumor volume reached an average size of 50–100 mm^3^ (*n* = 6 per group). The isolated tumors were photographed. **g** Tumor volume of each group was measured twice a week. After 21 days, the mice were sacrificed. The data were presented as mean ± SEM. **h** Immunohistochemistry was used to detect the expression of HIF-1α and Ki67 in the xenografts after various treatments. **i** Graphical summary of the molecular mechanisms involving MYH9, HIF-1α and USP22 in regulating of CSC properties in lenvatinib-resistant cells. The graph was created by Microsoft Powerpoint. The Student’s *t*-test was used for comparisons. *p < 0.05, ***p < 0.001. Scale bar: 100 μm. MYH9 non-muscle myosin heavy chain 9, LR lenvatinib resistant, HIF-1α hypoxia-inducible factor-1α, USP22 ubiquitin-specific protease 22, DMSO dimethyl sulfoxide, CSC cancer stem cell

Because USP22 is an important deubiquitinating enzyme for HIF-1α^9^, we speculated that p-MYH9 (Ser1943) might recruit USP22 to deubiquitinate and stabilize HIF-1α. To this end, we first demonstrated that USP22 knockdown could abrogate high MYH9-induced HIF-1α upregulation and promote ubiquitin-mediated degradation of HIF-1α in the HCC parent type or LR cells (Fig. 7a, b). Additionally, the endogenous interaction between MYH9, HIF-1α and USP22 was confirmed in HuH-7-LR cells (Fig. 7c). Furthermore, we performed co-IP assays in HEK-293T cells and found that MYH9 could interact with USP22, especially MYH9-S1943E, but not MYH9-S1943A (Fig. 7d; Supplementary Fig. 7a). That suggested that p-MYH9 (Ser1943) could specifically recruit USP22 to deubiquitinate HIF-1α. Finally, we evaluated the efficacy of USP22 inhibition in reversing LR. USP22 knockdown or USP22 inhibition effectively promoted the sensitivity of HCC LR cells and primary HCC cells to lenvatinib (Fig. 7e and Supplementary Fig. 7c). In vivo, the USP22 inhibitor (S02) significantly reversed LR in HuH-7-LR cells (Fig. 7f, g). The weights were not significantly different (Supplementary Fig. 7b). The expression levels of HIF-1α and Ki67 were further decreased in the S02 groups (Fig. 7h). Interestingly, S02 had little effect on lenvatinib sensitivity in HuH-7 cells (Supplementary Fig. 7d–f), which further supported the significant role of USP22 in HCC LR cells. The above results indicated that p-MYH9 (Ser1943) could recruit USP22 to deubiquitinate HIF-1α and promote LR (Fig. 7i).

## Discussion

Lenvatinib is approved by regulatory authorities worldwide as a first-line treatment for unresectable advanced HCC based on the positive results of the REFLECT trial.^4^ However, a previous study showed that over 60% of HCC patients who received lenvatinib monotherapy progressed within one year.^27^ Drug resistance is a main factor that limits the effectiveness of lenvatinib. There are several mechanisms that can contribute to LR, including genetic mutation,^8^ alternative pathway activation, tumor microenvironment (TME) changes,^28^ and cancer stem cell expansion. Exploring promising strategies to reverse LR and benefit more HCC patients is still a great challenge.

Cancer stemness is responsible for drug resistance, including cytotoxic drugs and TKIs.^29,30^ Recent studies have demonstrated that liver CSCs determine the response to lenvatinib treatment, with HIF-1α being a master driver.^31^ Most research on HIF-1α has focused on its role and effects in hypoxia, but the underlying mechanisms in normoxia have recently gained increasing attention.^32^ In leukemia and lymphoma, HIF-1α regulates cancer stemness in a hypoxia-independent manner.^22^ In breast cancer, HIF-1α mediated by G-protein coupled estrogen receptor (GPER) upregulated aerobic glycolysis and promoted tamoxifen resistance.^23^ Circulating tumor cells (CTCs) are suggested to originate from CSCs, circulating in the bloodstream rich in oxygen and being able to seed and colonize a second tumor in distant sites, which plays an important role in tumor metastasis, recurrence and drug resistance.^33^ A previous study showed that HIF-1α promoted the intravasation and metastasis of CTC clusters.^24^ Therefore, HIF-1α could be a promising target for the treatment of LR cells and CSCs.

MYH9, is a subunit of non-muscle myosin II, acting as a skeleton-related protein. MYH9 mutation leads to MYH9-related disease (MYH9-RD), which is a blood disease clinically manifested by thrombocytopenia with giant platelets.^34^ In cancers, the role of MYH9 as a tumor suppressor gene or an oncogene is controversial.^35^ MYH9 participates in cancer development by performing various functions, such as a transcriptional factor and a deubiquitinase.^26^ Thus, the mechanisms by which MYH9 is involved in HCC stemness and LR are meaningful and interesting. Recent studies have focused on the role of MYH9 phosphorylation in cancer progression.^36,37^ It has been identified that phosphorylation of MYH9 by protein kinase C (PKC) on Ser1916 and CK2 on Ser1943 is related to poor prognosis and could be a therapeutic target.^26^ In this study, HIF-1α was found to be upregulated in HCC LR cells and promoted cancer stemness and glycolysis even in normoxia. p-MYH9 (Ser1943) was first discovered to be increased in HCC LR cells and promoted LR and stemness in HCC cell lines and patient-derived HCC models. For a better understanding of the mechanism between p-MYH9 (Ser1943) and HIF-1α, we conducted in-depth research. USP22, a member of the deubiquitinating enzymes (DUBs) family, is a part of the Spt-Ada-Gcn5 acetyltransferase (SAGA) complex. USP22-mediated HIF-1α stabilization increased the cancer stemness of HCC cells under hypoxia.^9^ We found that USP22 knockdown could abrogate the HIF-1α expression induced by MYH9 and promote ubiquitin binding to HIF-1α. It was p-MYH9 (Ser1943) that recruited USP22 to decrease HIF-1α ubiquitination. This is further evidence that USP22 can collaborate with other proteins to play a crucial role in the progression of HCC, whether in hypoxia or normoxia.^38^

Silmitasertib (CX-4945), an oral CK2 inhibitor, has been approved by the FDA for cholangiocarcinoma (CCA) as an orphan drug. The application of CX-4945 effectively inhibited the development of CCA and suppressed HIF-1α expression.^39^These findings highlight the potential of CX-4945 as a novel therapeutic agent for clinical HCC patients. S02 is a compound selected from a small molecule inhibitor library and can effectively suppress USP22’s catalytic functions.^40^ While the provided data reveal one of the mechanisms between p-MYH9 (Ser1943) and HIF-1α, USP22 inhibition holds great therapeutic potential and the discovery of S02 offers the possibility of clinical application. Further clinical trials are needed to assess the safety and synergistic efficacy of CX-4945, S02 combined with lenvatinib in LR patients. Moreover, HIF-1α is constitutively ubiquitinated and degraded by E3 ubiquitin ligases but can be stabilized by various DUBs. Other DUBs, such as USP20, USP7, USP19 and USP28, have been reported to regulate the deubiquitination of HIF-1α.^41^ It is possible that there are additional mechanisms that have not yet been identified, including whether p-MYH9 (Ser1943) could impact the expression of USP22. A more comprehensive and detailed understanding of this topic would require a deeper exploration. By incorporating molecular typing and imaging omics into research, more advanced models can be created to predict LR in clinical settings.^42^

In conclusion, our research elucidates the mechanism of MYH9 and the p-MYH9 (Ser1943)/USP22/HIF-1α axis in promoting cancer stemness and LR in HCC. Targeting p-MYH9 (Ser1943) and USP22, CX-4945 and S02, respectively, can abolish HIF-1α-induced LR, presenting a potentially effective therapeutic option for individuals with HCC and LR.

## Materials and methods

### Cell culture and reagents

Human liver cancer cell lines (SNU-387, SNU-449, SNU-182, PLC/PRF/5, HLE, HepG2, MHCC-97H, MHCC-97L, Li-7, HuH-7 and Hep-3B) and HEK-293T were purchased from the Shanghai Institute of Cell Biology, Chinese Academy of Sciences (Shanghai, China). The verification of all cells’ authenticity was confirmed through short-tandem repeat (STR) profiling. The cells were cultured in an appropriate medium with 10% fetal bovine serum and 1% penicillin-streptomycin (Solarbio) at 37°C and 5% CO_2_. Lenvatinib-resistant cell lines were constructed in appropriate medium with increasing doses of lenvatinib (Selleck) from 2 μM. The media was replaced every 48 hours for 3–6 months and the resistant concentration was measured. The resistant cell lines were named HuH-7-LR, Hep-3B-LR and SNU-387-LR. To maintain the resistance, HuH-7-LR and Hep-3B-LR cells were cultured in the medium with 5 μM lenvatinib, and SNU-387-LR was with 10 μM lenvatinib. Li-7, SNU-387, SNU-182, SNU-449 and HLE cells were cultured in RPMI 1640 medium (Biological Industries). Hep-3B and HepG2 were maintained in MEM medium (Biological Industries). HEK-293T, HuH-7, MHCC-97H, PLC/PRF/5 and MHCC-97L cells were in DMEM medium with high glucose. The reagents employed in this research can be found in Supplementary Table 1.

### Patients and clinical specimens

A total of 171 patients who underwent resection for HCC were from the First Affiliated Hospital of Zhejiang University School of Medicine between 2015 and 2018. All patients were pathologically confirmed to have HCC and received ethical approval from the hospital’s Ethics Committee (Reference number: 20230733; 2018768). Additionally, 70 patients who received lenvatinib treatment were from Zhejiang Cancer Hospital and were pathologically confirmed to have HCC between 2020 and 2023. The study received ethical approval from the hospital’s Ethics Committee (Reference number: IRB-2022-508; IRB-2022-361).

### Plasmids transfection and lentivirus infection

Plasmids were purchased from Shandong Vigene and REPOBIO. The harvested DNA was inserted into pcDNA3.1-flag and pLVX-PURO-myc vectors by BamHI and XhoI restriction enzymes. Short hairpin RNAs (shRNA) against and overexpression of MYH9, HIF-1α and USP22 were packaged into lentiviral plasmids. No off-target effect was found in 4 in 1 plasmids of HIF-1α and USP22, according to a previous study.^9^ Site-directed mutagenesis of MYH9 and HIF-1α was performed according to reference.^26,43^ All oligonucleotide sequences are provided in Supplementary Table 2. Lentiviral particles were used to infect the cells with Lipofectamine 3000 (Invitrogen) according to manufacturer’s instructions. Cells with stable gene expression were selected using 5 μg/mL puromycin (Selleck). The reagents used in this study are shown in Supplementary Table 1.

### Cancer cell stemness

The stemness of HCC cells was determined by cell viability, colony formation, cell migration, cell sphere formation, drug resistance, cell counting and stemness gene expression assays. The detailed processes are provided in Supplementary Materials and the raw data of colony formation is provided in Supplementary Fig. 8.

### Glycolysis stress assay

The ECAR was assessed to determine the glycolytic capacity. The change in pH in the extracellular media surrounding cells over time was recorded by Seahorse XF Analyzers (Agilent). Therefore, the glycolytic capacity was calculated based on the parameters of the Wave data.

### Luciferase reporter assay

3 × 10^3^ cells were seeded on 6-well plates overnight, transfected with 50 ng pGL3-HRE-luciferase plasmid and 10 ng pSV40-renilla plasmid. Dual-luciferase reporter system was performed (Promega) to evaluate the HIF-1α transcriptional capacity. The results were determined by calculating the ratio of HRE luciferase activity to renilla activity. The experiment was repeated independently three times.

### Quantitative PCR (qPCR) analysis

Total RNA was extracted using the RNeasy Mini Kit (QIAGEN). The cDNA was generated using the Hiscript ® II RT SuperMix for qPCR (+gDNA wiper) kit and then amplified with ChamQ SYBR Color qPCR Master Mix (Vazyme). Real-time Quantitative PCR Detection was performed using the ABI 7500 Real-Time PCR System (Applied Biosystems). The sequences of primers are provided in Supplementary Table 3. The quantity of target cDNA was assessed by the converting the threshold cycle (CT) and normalizing it to β-actin. Each ample had three replicates.

### RNA-seq, KEGG analysis and gene set enrichment analysis

RNA-seq and data analysis were performed by RiboBio. Total RNA was extracted from cells, and mRNA was subsequently isolated using a poly(A) selection method. Differential expression analysis was conducted using DESeq2 with a significance level set at *p* < 0.05 and a fold change of threshold of >1.5. Cluster analysis was employed to investigate the expression profiles of different genes between LR cells and parent cells. The accessions for these SRA data are PRJNA1130017 and PRJNA11397.

### Co-IP assay, western blot analysis and ubiquitination assay

Cells were gathered, lysed and incubated according to previous study.^9^ The formulation of lysis buffers is provided in Supplementary Table 1. The detailed processes and methods are provided in Supplementary Materials. Antibodies and dilute concentrations are shown in Supplementary Table 4.

### Silver stain and mass spectrometry analysis

10^7^ cells were harvested, lysed and incubated with an appropriate amount of HIF-1α antibody as described above. Following immunoprecipitation, the bound proteins were isolated using SDS-PAGE and identified using silver staining. After excising and destaining the silver-stained proteins, mass spectrometry was carried out using the Pierce™ Silver Stain for Mass Spectrometry kit (Thermo). The samples were then analyzed by LC/MS. The accession for Mass Spectrometry data is PXD054805.

### Flow cytometry detection

1.5 × 10^5^ cells were seeded onto a 6-well plate and collected post-treatment. The cells were resuspended in Annexin V Binding Buffer and stained with PI Solution and FITC (Dojindo). The incubation was performed on ice for 30 minutes in the dark. The analysis was conducted using a FACSCanto II analyzer flow cytometer (BD Biosciences).

### Immunofluorescence

5 × 10^4^ cells were seeded on glass coverslips attached to 6-well plates. After being cultured for 24 hours, the coverslips were fixed with 4% paraformaldehyde, washed with PBS and blocked with goat serum. Diluted primary antibodies were added to the coverslips and incubated at 4°C overnight. Then, appropriate fluorochrome-conjugated secondary antibodies were added and incubated at room temperature in the cassette for 2 hours. DAPI (Beyotime) staining was then employed to indicate localization of nuclear. The distribution of the various fluorescence signals was examined with a laser-scanning confocal microscope (Olympus).

### Animal experiments

Animal care and experiments were conducted in strict accordance with the ‘Guide for the Care and Use of Laboratory Animals’ and the ‘Principles for the Utilization and Care of Vertebrate Animals’. Approval was obtained from the Medical Ethics Committee of the First Affiliated Hospital of Zhejiang University, School of Medicine (Reference number: 20231341). An appropriate number of HCC cells were subcutaneously planted into the mice and administrated with drugs after the tumor reached a certain size. The mice were sacrifice and the tumors were isolated for further research. For orthotopic tumor model, the HCC cells were injected into the sub-capsular space of right liver lobe on eight weeks male nude mice. For PDX, HCC tumor tissue was acquired from patients by surgery, divided into 20–30 mm^3^ pieces and transplanted into subcutaneously five-week-old NOD/SCID mice.

Detailed processes and methods of subcutaneous tumor model, HCC orthotopic tumor model and PDX are described in Supplementary Materials.

### Primary HCC cells and PDO

Human HCC tumor tissue was acquired from patients by surgery. Primary HCC cells were isolated and used in further experiments. Detailed processes and methods are described in Supplementary Materials.

### Immunohistochemistry

HCC samples were embedded in paraffin, following the steps of sectioning, dewaxing, antigen retrieval, blocking and immunostaining according to the Immunohistochemical assay kit (Proteintech). The tissues were scanned by a microscope and evaluated by staining intensity and positive area ratio. The immunohistochemical score (IS) was calculated by multiplying the intensity score by the percentage score. The tissues with IS >7 were identified as high expression. The gradations are ranked as shown in Supplementary Table 5. The antibodies used for immunohistochemistry are listed in Supplementary Table 4.

### Statistical analysis

SPSS Statistics 22 software and GraphPad Prism software were used for the statistical analysis. The student’s *t*-test was employed for comparing two groups, and the chi-square test was used for correlation analysis. The Kaplan-Meier method was utilized for survival analysis, with significance indicated by *p* < 0.05.

## Supplementary information

Supplementary_Materials

## Data Availability

RNA-sequencing data has been uploaded to the SRA database (PRJNA1130017& PRJNA1131297). Mass Spectrometry data has been uploaded to iProX (IPX0009565000). All of data supporting the findings is present in the paper and can be obtained from the corresponding author upon reasonable request.
