# Supplementary material for: The p-MYH9/USP22/HIF-1α axis promotes lenvatinib resistance and cancer stemness in hepatocellular carcinoma
Source: Signal Transduct Target Ther. 2024 Sep 19;9:249. doi: 10.1038/s41392-024-01963-5 (PMC11412978; doi:10.1038/s41392-024-01963-5)
Supplement: Supplementary file 1 — Supplementary_Materials [file 41392_2024_1963_MOESM1_ESM.docx]

Supplementary Materials for

The p-MYH9/USP22/HIF-1α axis promotes lenvatinib resistance and cancer stemness in hepatocellular carcinoma

#Qiaonan Shan, #Lu Yin, #Qifan Zhan, #Jiongjie Yu, Sheng Pan, Jianyong Zhuo, Wei Zhou, Jiaqi Bao, Lincheng Zhang, Jiachen Hong, Jianan Xiang, Qingyang Que, Kangchen Chen, Shengjun Xu, Jingrui Wang, Yangbo Zhu, Bin He, Jingbang Wu, Haiyang Xie, Shusen Zheng, *Tingting Feng, *Sunbin Ling, *Xiao Xu

Correspondence to: Xiao Xu ( [zjxu@zju.edu.cn](mailto:zjxu@zju.edu.cn) ); Sunbin Ling ( [lsb0330@zju.edu.cn](mailto:lsb0330@zju.edu.cn) ); Tingting Feng ( [fengtt@zjcc.org.cn](mailto:fengtt@zjcc.org.cn) ).

This PDF file includes:

Supplementary Materials and Methods

Figures. S1 to S8

Tables S1 to S7

**Supplementary Materials and Methods**

**Cell viability assay**

3 x 10^3^ cells were seeded on 96-well plates and incubated overnight. The next day, the medium was replaced with fresh medium containing DMSO or a gradient concentration of lenvatinib. After 72 h, the medium was exchanged with 100 μL of RPMI-1640 and 10 μL of CCK-8 reagent for an additional 3 h at 37˚C. Subsequently, the optical density was measured at 450 nm with an EnSpireTM 2300 Multilabel Reader (PerkinElmer). Every treatment has five replicates. IC50 values were determined by GraphPad Prism 8.0 using a 3-parameter dose–response model.

**Colony formation assay**

Cells were seeded on 12-well plates in triplicate at a density of 2 x 10^3^ cells per well. The next day, the medium was replaced with fresh medium containing DMSO or the indicated treatment. After being cultured for 2 weeks, the clones were rinsed, fixed with 1% paraformaldehyde and stained with crystal violet solution (Beyotime). The number of clones was visually counted.

**Cell migration assay**

3 x 10^4^ cells were seeded on a polycarbonate membrane in the upper compartment of a chamber (Corning Incorporated) with 200 μL FBS-free medium. 800 μL medium with 15% FBS was added to the lower compartment of the chamber. After 24 h incubation, the cells that had migrated through the membrane were washed, fixed with 1 % paraformaldehyde and stained with crystal violet solution. Photographs of 3 randomly selected fields of the fixed cells were captured and cells were counted. The experiments were repeated independently three times.

**Cell sphere formation**

2 x 10^3^ cells were plated on 6 well plates with Ultra Low Attachment surface (Corning Incorporated) and cultured with special medium containing DMEM/F12 medium (Invitrogen) supplemented with 4 μg/mL insulin (Sigma-Aldrich), B27 (Invitrogen), 20 ng/mL EGF (Sigma-Aldrich), and 20 ng/mL basic FGF (Invitrogen). After incubation for 10 days, the number of spheres with diameters of more than 75 µm was photographed and counted by microscope.

**Cell counting**

3 x 10^4^ cells were planted on 12-well plates overnight. The next day, the cultures were replaced with fresh medium containing DMSO or the indicated treatment. After cultured for 48 h, the cells were digested into a single cell suspension, stained with a 1% trypan blue solution (Beyotime) and counted by the cell counting chamber under the microscope. Every treatment had three replicates.

**Co-IP assay**

10^7^ cells were harvested and lysed with IP Lysis Buffer containing protease or phosphatase inhibitors (Thermo Fisher) according to previous study^[1]^. A small amount of lysate was used for western blot analysis as input. 20 μL Protein A/G agarose beads (Thermo Fisher) and 1.5 μg primary antibody were added to the cell supernatant and incubated at 4˚C for 2- 4 h. The antigen-antibody complex binding to beads was centrifuged and eluted using Elution buffer (Thermo Fisher) according to instructions. Immunoprecipitated proteins were separated and detected by western blot analysis.

**Western blot analysis**

The proper number of cells were collected and subsequently lysed using RIPA Lysis Buffer. The protein lysates were quantified by BCA protein analysis kit (Thermo Fisher), separated by SDS-PAGE and transferred to a polyvinylidene fluoride membrane. The membrane was incubated at 4℃ with the primary antibody overnight and then with HRP-conjugated secondary antibodies at room temperature for 1 h. The bands were visualized by chemiluminescence.

**Ubiquitination assay**

Cells were harvested and lysated in a buffer containing 50 mM Tris (pH 7.4), 150 mM NaCl, 1% NP-40, 0.5% sodium deoxycholate, and 2% SDS. Before immunoprecipitation, the lysates were boiled at 95℃ for 5 min. Immunoprecipitation and western blot analysis were conducted as described above.

**Animal experiments**

Subcutaneous tumor model

To test the in vivo tumor initiating capacity, a limiting dilution assay was performed. Different numbers of HuH-7 cells and HuH-7-LR cells were subcutaneously injected into five-week-old nonobese diabetic/severe combined immunodeficiency (NOD/SCID) mice. 4 weeks later, the mice were sacrificed and the tumors were isolated. ELDA software was used to analyze stemness. In athymic nude mouse models, 2 x 10^6^ HuH-7 or HuH-7-LR cells were subcutaneously injected into five-week-old male mice. After the tumor volume reached an average size of 50-100 mm^3^, mice were divided into groups according to experimental requirements. Lenvatinib, CX4945, S02 were suspended in 0.5% sodium carboxymethyl cellulose (CMC) (prepared with distilled water). 100 μL 0.5% CMC was daily used as vehicle group. 100 μL lenvatinib (30 mg/kg/day, gavage), CX-4945 (20 mg/kg/day, gavage) and S02 (10 mg/kg/day, gavage) were daily used as experimental groups. HuH-7 and HuH-7-LR xenografts treated with 0.5% CMC or lenvatinib were used to evaluate the lenvatinib resistance changes in vivo. To test the effect of CX-4945 and S02, HuH-7-LR xenografts were used. The tumor size and mice weight were observed and measured twice a week. After three weeks, all the mice were sacrificed. The tumors were photographed and the volume was calculated by width^2^ x length x 1/2. Consistent with the guidelines and principles, all mice were euthanized as soon as measurements indicated that the tumors reached the size limit approved (1.5 cm). Given the soft nature of the tumor, there are inherent inaccuracies in subcutaneous measurements and image presentation.

HCC orthotopic tumor model

A total of 40 μL of PBS containing HuH-7-LR cell (2 x 10^6^) and basement membrane matrix (1:1) was injected into the sub-capsular space of right liver lobe on eight weeks male nude mice to establish the orthotopic tumor model. Mice were randomly assigned to each group. After 1 week of tumor growth in orthotopic model, mice were treated with vehicle, CX-4945 (20 mg/kg, gavage), lenvatinib (30 mg/kg, gavage) and their combination daily for 3 weeks. Each animal was earmarked and followed individually throughout the experiment. Tumor volume was calculated with formula: length x width ^2^ x 1/2 (mm^3^). At day 28th, mice were sacrificed and tumors were photographed.

PDX

Human HCC tumor tissue was acquired from patients by surgery. Fresh tissues were rinsed with PBS and divided into 20-30 mm^3^ pieces. The tumor tissues were subcutaneously transplanted into five-week-old NOD/SCID mice. After the tumor volume reached an average size of 50-100 mm^3^, mice were divided into groups and daily treated with vehicle (0.5% CMC) or lenvatinib (30 mg/kg, gavage). After two weeks, all the mice were sacrificed. The tumors were photographed and the volume was calculated by width^2^ x length x 1/2.

**Primary HCC cells and PDO**

Fresh resected tissues were minced, rinsed with PBS, and incubated in digestion buffer containing 2 mg/mL collagenase D (Worthington) on an orbital shaker at 37 °C. Incubation time of the specimen was dependent on the amount of collected tissue and ranged from 30-90 min, until the majority cell clusters were in suspension. After tissue digestion, DMEM media containing 10% FBS was added to the suspension to inactivate collagenase D and cell suspension was then filtered through a 70 μm Nylon cell strainer and centrifuge 5 min at 300 g, 4 ℃. During processing, cell suspension could be counted by Trypan Blue to determine the concentration of live cells. The primary HCC cells were used in further experiments. To culture PDO, all the pellets were washed in cold PBS twice and kept cold. 5,000-10,000 isolated cells mixed with cold Matrigel Basement Membrane Matrix (bioGenous) and 50 μL drops of Matrigel-cell suspension were allowed to solidify on prewarmed 24-well suspension culture plates at 37°C for 30 min. Upon completed gelation, 500 μL of HCC organoid medium (bioGenous) was added to each well and plates transferred to humidified 37 °C /5% CO_2_ incubators at either 2% or ambient O_2_. Medium was changed every 3-4 days and organoids were passaged every 1- 4 weeks.Organoid cultures are usually passaged with a split ratio of 1:3 every 2-3 weeks by organoid dissociation solution (bioGenous) and re-seeded into new Matrigel. HCC organoids were fixed in a 10% formalin solution for 1 h at room temperature, and embedded in paraffin. Sections (3 μm-thick) were taken and stained with hematoxylin and eosin (H&E). HCC organoids were scanned by a microscope.


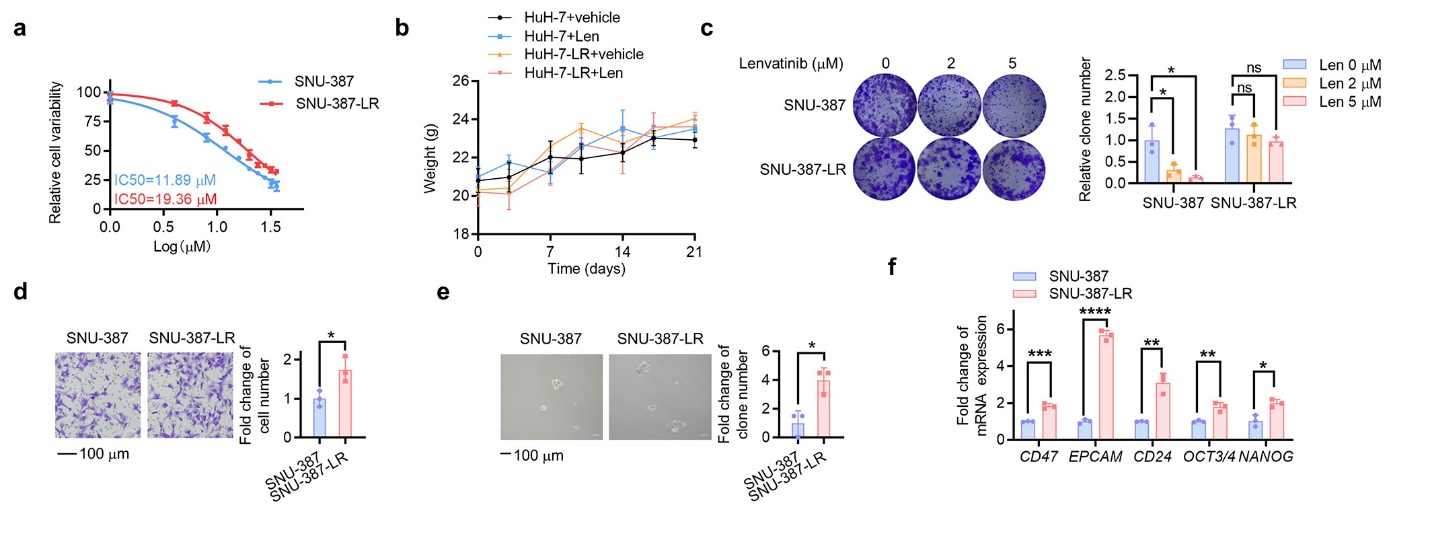


**Supplementary Fig. 1.** Acquired lenvatinib-resistant SNU-387 cells display increased cancer stemness. **a** The IC50 value of SNU-387 and SNU-387-LR cells was determined using the CCK-8 assay. Each point on the dose-response curves represents five technical replicates. **b** The mouse weight of each group (n = 5 per group) in Fig 1**c** was measured twice a week. The cancer cell stemness of SNU-387 and SNU-387-LR cells was evaluated through colony formation (**c**), migration (**d**), in vitro self-renewal (**e**), and the mRNA expression of stemness markers (**f**). Scale bar: 100 μm. Histograms were used to statistically analyze changes in colony formation in SNU-387 and SNU-387-LR cells. The data of cell functional assays and RT-qPCR assay were presented as mean ± SD of three individual experiments, and the data of animal experiments were presented as mean ± SEM. Comparisons were performed with Student's *t*-test. **p*<0.05, ***p*<0.01, ****p*<0.001, *****p*<0.0001. LR, lenvatinib resistant; CCK-8, Cell Counting Kit-8; ns, nonsignificant.


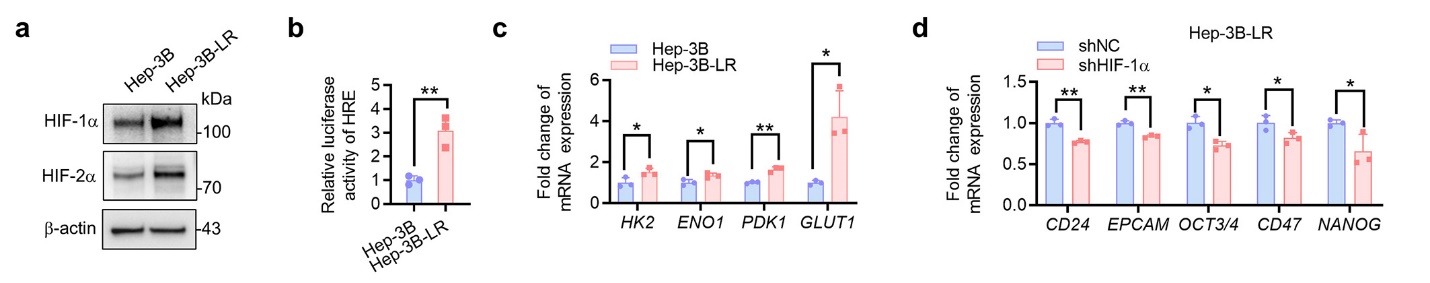


**Supplementary Fig. 2.** HIF-1α pathway is activated in Hep-3B-LR cells and promotes cancer stemness. **a** The protein expression of HIF-1α and HIF-2α in Hep-3B and Hep-3B-LR cells. **b** Fold change of the relative luciferase activity was examined by luciferase reporter assay in Hep-3B and Hep-3B-LR cells. **c** The mRNA expression of glycolysis driver genes in Hep-3B and Hep-3B-LR cells was measured by qRT‒PCR assay. **d** The mRNA expression of stemness markers was measured by qRT‒PCR in Hep-3B-LR cells with HIF-1α knockdown (shHIF-1α) or not (shNC). Data were presented as mean ± SD of three individual experiments. Comparisons were performed with Student's *t*-test. **p*<0.05, ***p*<0.01. HIF-1α, hypoxia-inducible factor-1α; HIF-2α, hypoxia-inducible factor-2α; LR, lenvatinib resistant.


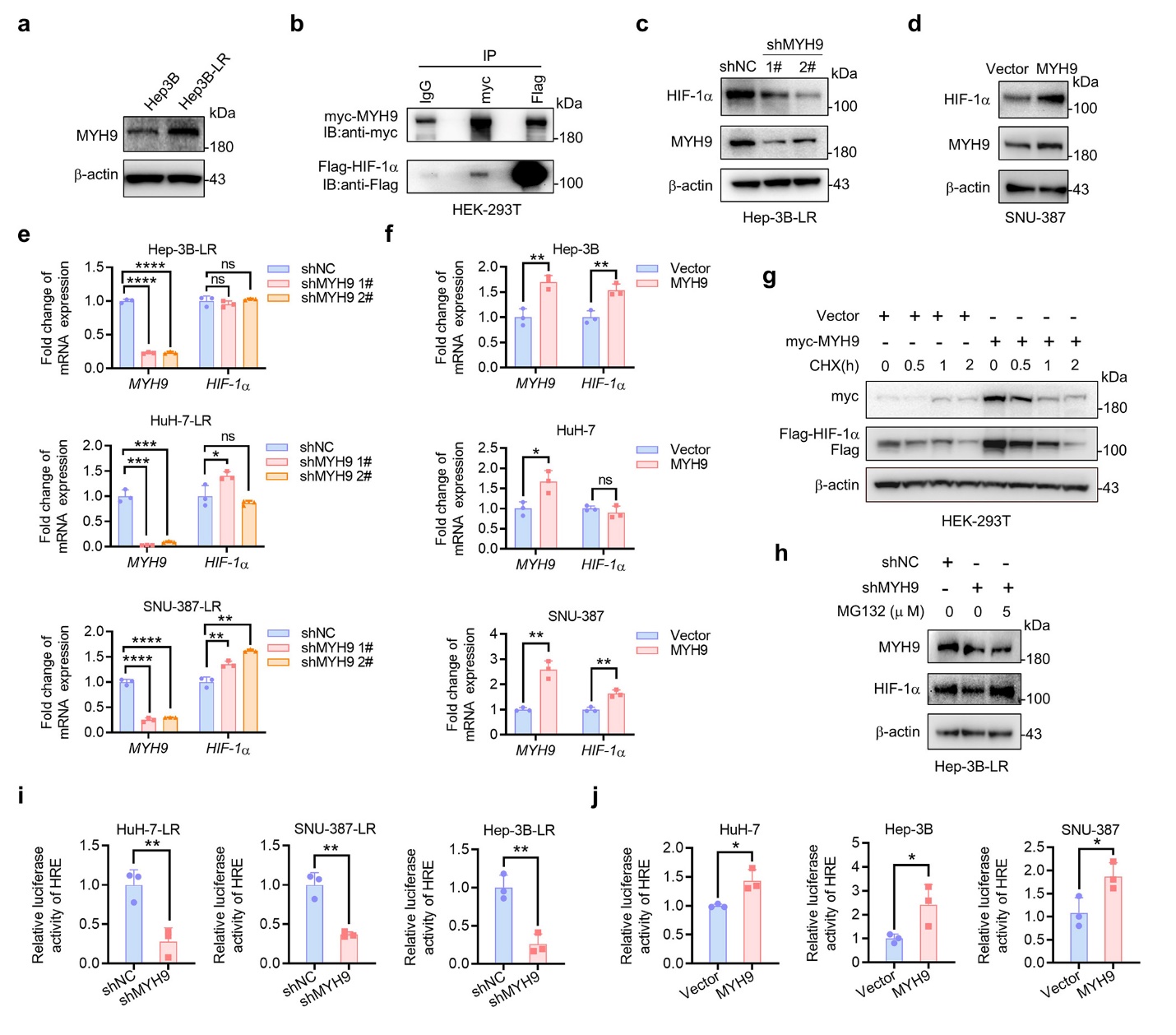


**Supplementary Fig. 3.** MYH9 inhibits the degradation of HIF-1α by ubiquitination. **a** The expression of MYH9 in Hep-3B and Hep-3B-LR cells was detected using western blot. **b** The interaction between exogenous MYH9 and HIF-1α was tested in HEK-293T cells. Normal rabbit IgG was used as a control. **c** The protein expression of MYH9 and HIF-1α in Hep-3B-LR cells with MYH9 knockdown (shMYH9) or shNC was detected using western blot analysis. **d** The protein expression of MYH9 and HIF-1α in Hep-3B cells with MYH9 overexpression (MYH9) or vector was detected using western blot analysis. **e** The mRNA expression of MYH9 and HIF-1α in the HCC cells with MYH9 knockdown (shMYH9 1# and 2#) or not (shNC) were detected by qRT‒PCR assay. **f** The mRNA expression of MYH9 and HIF-1α in the HCC cells with MYH9 overexpression (MYH9) or not (vector) were detected by qRT‒PCR assay. **g** HEK-293T cells with MYH9 overexpression (MYH9) or vector were treated with 10 μg/mL CHX for the indicated time course. The protein levels of exogenous HIF-1α and MYH9 were analyzed by western blot. **h** Hep-3B-LR cells with or without MYH9 knockdown (shMYH9 or shNC) were treated with or without MG132 (5 μM) for 2 hours. The protein levels of MYH9 and HIF-1α were analysed by western blot. (**i** and **j**) Fold change in the relative luciferase activity was examined by luciferase reporter assay in LR or WT HCC cells with MYH9 knockdown, overexpression or not. Data were presented as mean ± SD of three individual experiments. Comparisons were performed with Student's *t*-test. **p*<0.05, ***p*<0.01, ****p*<0.001, *****p*<0.0001. LR, lenvatinib resistance; WT, wide type. MYH9, nonmuscle myosin heavy chain 9; LR, lenvatinib resistant; HIF-1α, hypoxia-inducible factor-1α; HCC, hepatocellular carcinoma; CHX, cycloheximide; WT, wild type; ns, nonsignificant.

**
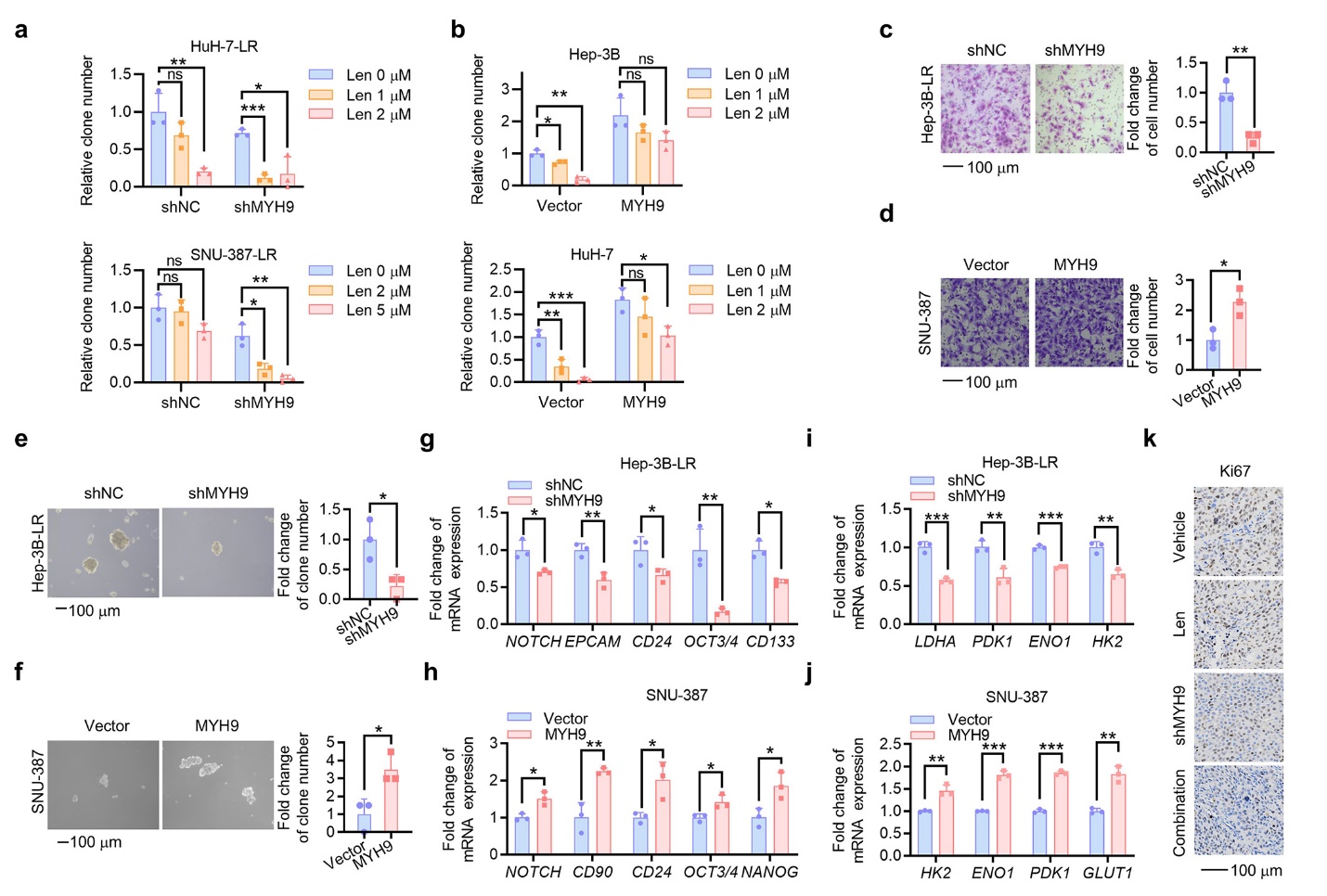
Supplementary Fig. 4.** MYH9 promotes LR and cancer stemness in HCC. (**a** and **b**) Histograms were used to statistically analyze changes in colony formation in Fig 4**a** and **b**, respectively. The effects of and MYH9 knockdown (shMYH9) or not (shNC) on Hep-3B-LR cell stemness shown according to migration (**c**), in vitro self-renewal (**e**), the mRNA expression of stemness markers (**g**) and glycolysis driver genes (**i**). The effects of MYH9 overexpression (MYH9) or not (vector) on SNU-387 cell stemness were shown according to migration (**d**), in vitro self-renewal (**f**), the mRNA expression of stemness markers (**h**) and glycolysis driver genes (**j**). **k** Expression of Ki67 in the HuH-7-LR xenografts after drug treatment was detected by IHC. Data were presented as mean ± SD of three individual experiments. Comparisons were performed with Student's *t*-test. **p*<0.05, ***p*<0.01, ****p*<0.001. Scale bar: 100 μm. MYH9, nonmuscle myosin heavy chain 9; LR, lenvatinib resistant; IHC, immunohistochemistry; ns, nonsignificant.

**
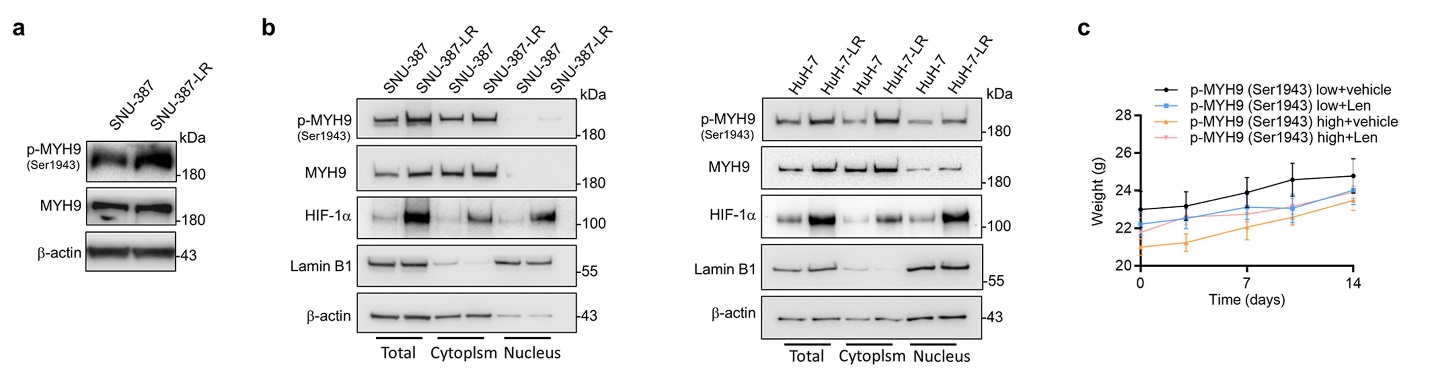
 Supplementary Fig. 5.** The expression and cellular location of p-MYH9(Ser1943), MYH9 and HIF-1α. **a** The expression of p-MYH9 (Ser1943) in SNU-387 and SNU-387-LR cells was detected using western blot. **b** The expression of p-MYH9 (Ser1943), MYH9, HIF-1α in whole cell lysate (total), cytoplasm or nucleus in wide type or LR HCC cells was detected using western blot. **c** The mouse weight of each group (n = 5 per group) in Fig 5**l** was measured twice a week. The data of animal experiments were presented as mean ± SEM. MYH9, nonmuscle myosin heavy chain 9; LR, lenvatinib resistance; HIF-1α, hypoxia-inducible factor-1α; HCC, hepatocellular carcinoma.


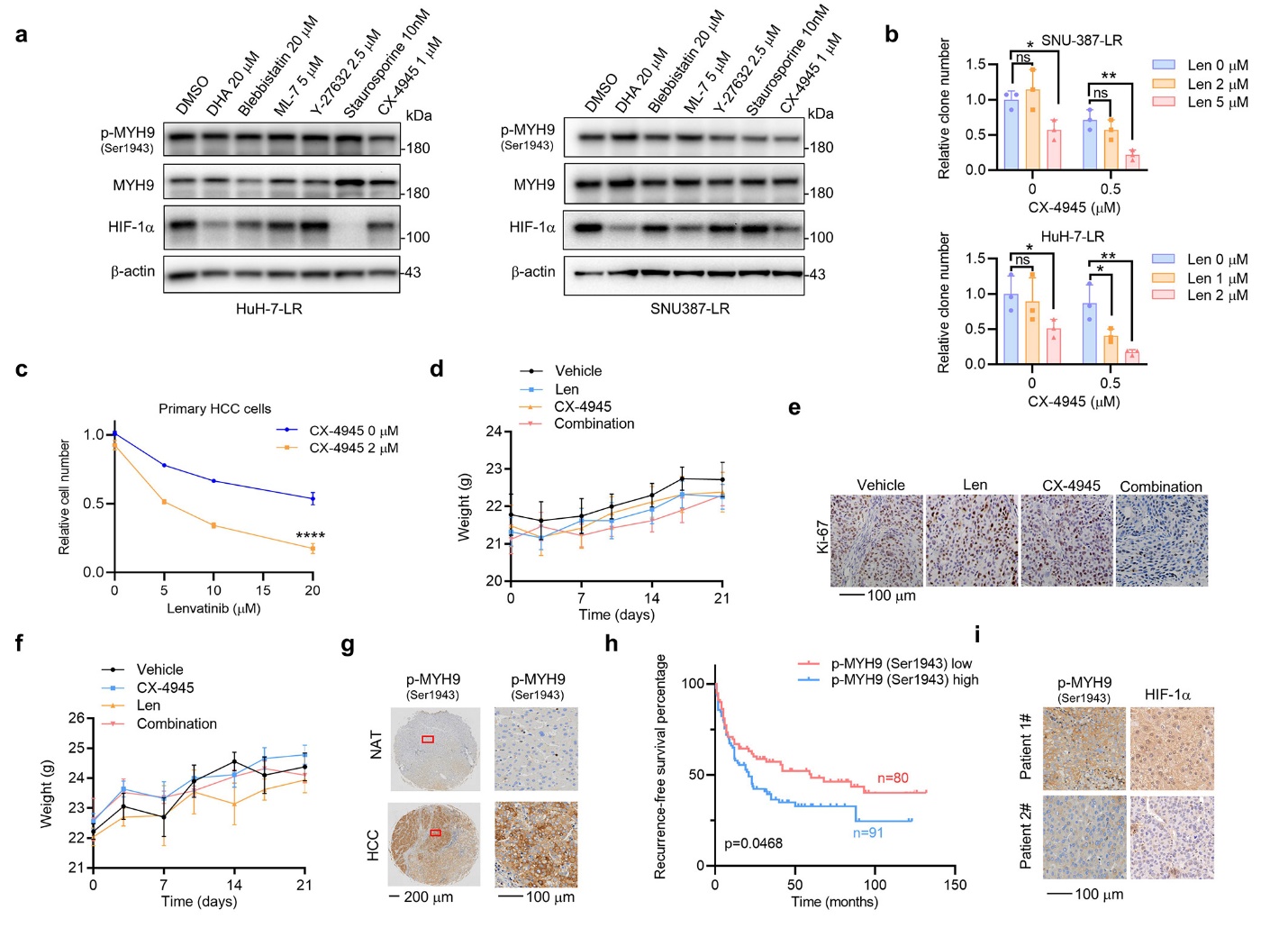


**Supplementary Fig. 6.** Targeting p-MYH9 (Ser1943) suppresses the expression of HIF-1α, reduces LR and improves prognosis. **a** In HCC LR cells with a series of MYH9-related inhibitors or DMSO treatment, the expression of p-MYH9 (Ser1943), MYH9 and HIF-1α was detected by using western blot. **b** Histograms were used to statistically analyze changes in colony formation in Fig 6**f**. **c** Primary HCC cells were treated with DMSO or 2 μM CX-4945 combined with the indicated concentration of lenvatinib. Variations in lenvatinib and CX-4945 sensitivity were detected by trypan blue staining-based cell count. **d** The mouse weight of each group in Fig 6**h** was measured twice a week, and (**e**) the Expression of Ki67 in the xenografts after treatment was detected by IHC. **f** The mouse weight of each group (n = 5 per group) in Fig 6**k** was measured twice a week. **g** The IHC images of p-MYH9 (Ser1943) for Fig 6**m**. **h** Recurrence free curves for 171 patients in Fig 6**n**. **i** The IHC images of p-MYH9 (Ser1943) and HIF-1α for Fig 6**p**. The data of cell functional assays were presented as mean ± SD of three individual experiments, and the data of animal experiments were presented as mean ± SEM. Comparisons were performed with student's *t*-test. **p*<0.05, ***p*<0.01, *****p*<0.0001. Scale bar: 100 μm. HCC, hepatocellular carcinoma; LR, lenvatinib resistance; MYH9, nonmuscle myosin heavy chain 9; DMSO, dimethylsulfoxide; HIF-1α, hypoxia-inducible factor-1α; IHC, immunohistochemistry; ns, nonsignificant.

**
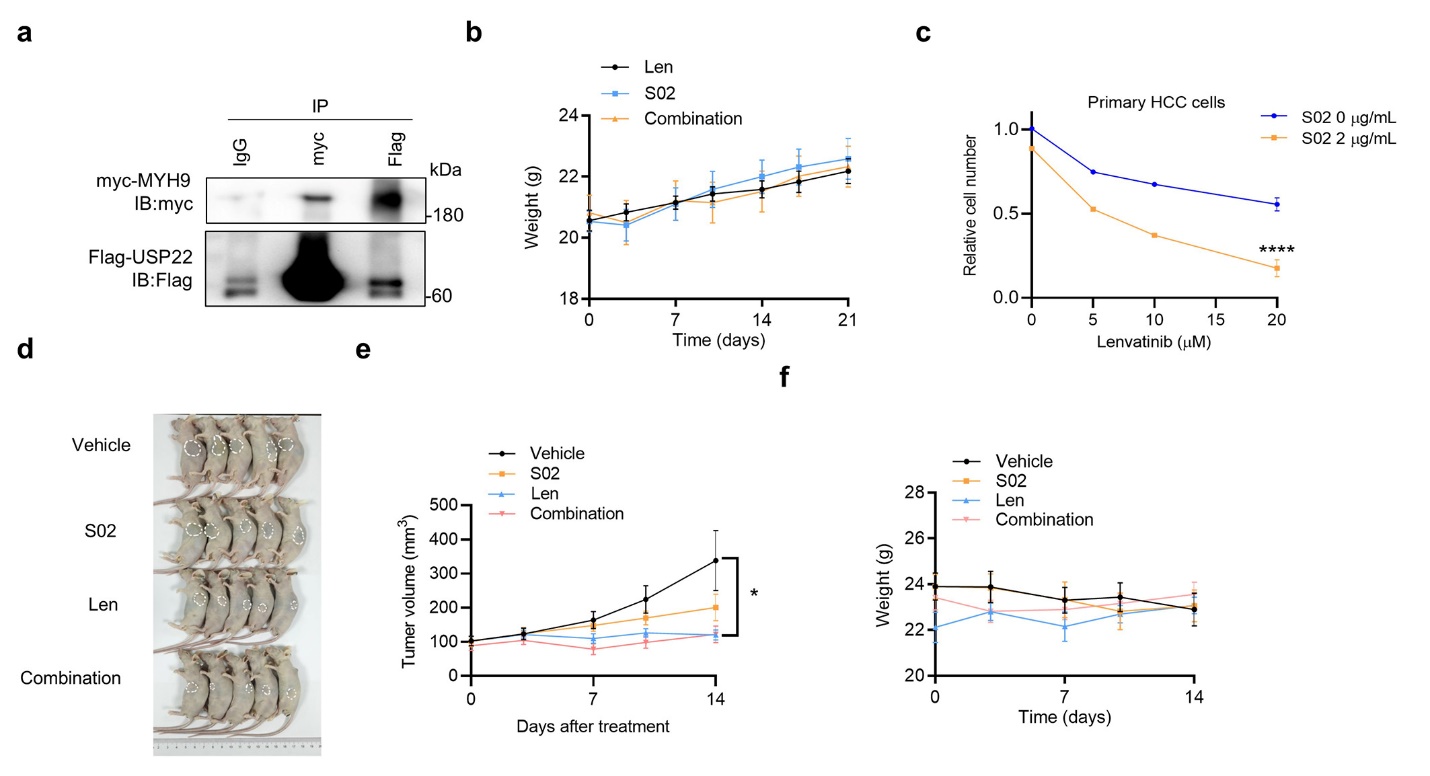
Supplementary Fig. 7.** USP22 interacts with MYH9 and promotes LR. **a** The interaction of exogenous USP22 and MYH9 was detected in HEK-293T cells with co-transfected USP22 (Flag labeled) MYH9 (myc labeled). Normal rabbit IgG was used as a control. **b** The mouse weight of each group (n = 6 per group) in Fig 7**f** was measured twice a week. **c** Primary HCC cells were treated with DMSO or 2 μg/mL USP22 inhibitor, S02 combined with the indicated concentration of lenvatinib. Variations in lenvatinib and USP22 inhibitor sensitivity were detected by trypan blue staining-based cell count. **d** HuH-7 cell xenografted nude mice were treated with vehicle, S02 (10 mg/kg), Len (lenvatinib, 30 mg/kg) and their combination for 2 weeks after the tumor reached an average size of 100 mm^3^ (n = 5 per group). Then, the mice were sacrificed when the diameters of one of xenografts reaches 1.5 cm and the mice were photographed. Tumor volume (**e**) and mouse weight (**f**) of each group was measured twice a week. The data of cell count were presented as mean ± SD of three individual experiments, and the data of animal experiments were presented as mean ± SEM. **p*<0.05, *****p*<0.0001. USP22, ubiquitin-specific protease 22; MYH9, nonmuscle myosin heavy chain 9.


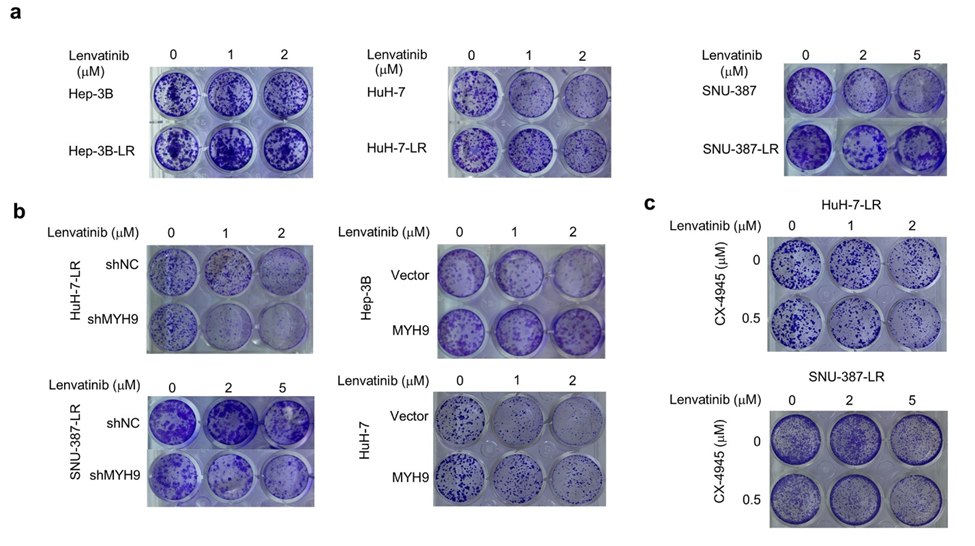


**Supplementary Fig. 8.** The raw data of colony formation. **a** The raw data of Fig.1**e** and Supplementary Fig. 1**c**. **b** The raw data of Fig. 4**a**. **c** The raw data of Fig. 6**f**.

**Supplementary Table 1. Detailed information of reagants used in experiments**

| Reagents | Company | Product code |
| --- | --- | --- |
| Penicillin-streptomycin | Solarbio | Cat#P1400 |
| Fetal bovine serum | WISIENT | Cat#086-150 |
| Lenvatinib (E7080) | Selleck | Cat#S1164 |
| Silmitasertib (CX-4945) | Selleck | Cat#S2248 |
| Usp22i-S02 | Selleck | Cat#E1330 |
| MG132 | Selleck | Cat#S2619 |
| Cycloheximide | Sigma-Aldrich. | Cat#508739 |
| Cobalt (II) chloride | Sigma-Aldrich. | Cat#409332 |
| Puromycin | Solarbio | Cat#P8230 |
| HiScript II Q RT SuperMix for qPCR (+gDNA wiper) | Vazyme | Cat#R223-01 |
| 2 × ChamQ Universal SYBR qPCR Master Mix | Vazyme | Cat#Q711-02 |
| DAPI solution | Solarbio | Cat#C0065 |
| IP Lysis Buffer  (50mM Tris (pH 7.4), 150 mM NaCl, 1% NP-40, 0.5% sodium deoxycholate, 0.1% SDS) | Thermo Fisher | Cat#87787 |
| RIPA Lysis buffer  (50mM Tris（pH 7.4）, 150 mM NaCl, 1%TritonX-100, 1% sodium deoxycholate, 0.1% SDS) | FDbio | Cat#FD009 |
| Hepatocellular Carcinoma Organoid Basal Medium | Biogenous | Cat#K2105-HCC |
| Collagenase Type 4 | Worthington biochemical | Cat#LS004188 |
| Lipofectamine 3000 | Invitrogen | Cat#L3000150 |
| RNeasy Mini Kit | QIAGEN | Cat#74104 |
| Annexin V, FITC Apoptosis Detection Kit | Dojindo | Cat#AD10 |
| Immunohistochemical assay kit | Proteintech | Cat#PK10006 |

**Supplementary Table 2. Detailed information of oligonucleotides used in experiments**

| Oligonucleotides | Company |
| --- | --- |
| 4in1 shRNA targeting sequence  HIF1α 1#  CGGCGAAGTAAAGAATCTGAA;  HIF1α 2#  TGATGAAAGAATTACCGAATT;  HIF1α 3#  CCGCTGGAGACACAATCATAT;  HIF1α 4#  CCAGTTATGATTGTGAAGTTA | ViGene |
| 4in1 shRNA targeting sequence  USP22 1#  GAGCTACCAGGAGTCCACAAAG;  USP22 2#  TGTGCCAGGACTACATCTATG;  USP22 3#  GCGAAGGGTACTTGCTGTTCTA;  USP22 4#  GCCTACCTGCTGTAAGATTAUG | ViGene |
| shRNA targeting sequence  MYH9 1#  GCAAGCUGCCGAUAAGUAU  MYH9 2#  GCAAAUUCAUUCGCAUCAA | REPOBIO |
| pLent-4in1shRNA-GFP-Puro-HIF1α | ViGene |
| pLent-4in1shRNA-GFP-Puro-USP22 | ViGene |
| pLKO.1-PURO-MYH9-shRNA-1 | REPOBIO |
| pLKO.1-PURO-MYH9-shRNA-2 | REPOBIO |
| pLVX-PURO-myc-MYH9 | REPOBIO |
| pLVX-PURO-myc-MYH9 S1943E | REPOBIO |
| pLVX-PURO-myc-MYH9 S1943A | REPOBIO |
| pLVX-PURO-myc-MYH9 S1916E | REPOBIO |
| pcDNA 3.1-flag-USP22 | REPOBIO |
| pcDNA 3.1-flag-HIF1α WT | REPOBIO |
| pcDNA 3.1-flag-HIF1α (P402A, P564A) | REPOBIO |
| pGL3-HRE-luciferase | REPOBIO |
| pSV40-renilla | REPOBIO |
| pLent-puro-HA-ubiquitin | REPOBIO |

**Supplementary Table 3. Detailed information of primers used in experiments**

| Gene | Forward primer (5’-3’) | Reverse primer (5’-3’) |
| --- | --- | --- |
| -actin | CATCCACGAAACTACCTTCAACTCC | GAGCCGCCGATCCACACG |
| NOTCH | CCTGAGGGCTTCAAAGTGTC | CGGAACTTCTTGGTCTCCAG |
| CD90 | GACAGCCTGAGAGGGTCTTG | CCCAGTGAAGATGCAGGTTT |
| CD24 | TGAAGAACATGTGAGAGGTTTGAC | GAAAACTGAATCTCCATTCCACAA |
| OCT3/4 | CTTGCTGCAGAAGTGGGTGGAGGAA | CTGCAGTGTGGGTTTCGGGCA |
| NANOG | AATACCTCAGCCTCCAGCAGATG | TGCGTCACACCATTGCTATTCTTC |
| EPCAM | AATCGTCAATGCCAGTGTACTT | TCTCATCGCAGTCAGGATCATAA |
| CD47 | GGCAATGACGAAGGAGGTT | ATCCGGTGGTATGGATGAGA |
| HK2 | GAGCCACCACTCACCCTACT | CCAGGCATTCGGCAATGTG |
| ENO1 | AAAGCTGGTGCCGTTGAGAA | GGTTGTGGTAAACCTCTGCTC |
| LDHA | TTGACCTACGTGGCTTGGAAG | GGTAACGGAATCGGGCTGAAT |
| GLUT-1 | GGCCAAGAGTGTGCTAAAGAA | ACAGCGTTGATGCCAGACAG |
| HIF1A | CGTGTTATCTGTCGCTTTGAGTC | GTCTGGCTGCTGTAATAATGTTCC |

**Supplementary Table 4. Detailed information of antibodies used in experiments**

| Antibodies | Company | Product code | Dilution |
| --- | --- | --- | --- |
| Beta Actin antibody | Proteintech | Cat# 60008-1-Ig | Western Blot: 1/10000 |
| Goat anti-mouse IgG (H+L) | Proteintech | Cat# SA00001-1 | Western Blot: 1/10000 |
| Goat anti-rabbit IgG (H+L) | Proteintech | Cat# SA00001-2 | Western Blot: 1/10000 |
| Anti-HIF-1 alpha | Abcam | Car#ab179483 | Western Blot: 1/2000 |
| Anti-non-muscle Myosin IIA | Abcam | Car#ab238131 | Western Blot: 1/2000 |
| HIF2 alpha antibody - ChIP Grade | Abcam | Cat# ab199 | Western Blot: 1/2000 |
| Monoclonal ANTI-FLAG® M2 antibody | Sigma-Aldrich | Cat# F1804 | Western Blot: 1/2000;  Immunoprecipitation: 1.5-2 μg for 1.0 mg of total protein lysate |
| Rabbit anti- Myc tag | Abcam | Cat#ab9106 | Western Blot: 1/2000;  Immunoprecipitation: 1.5-2 μg for 1.0 mg of total protein lysate |
| Anti-HA tag | Abcam | Cat#ab9110 | Western Blot: 1/5000 |
| Phospho-MYH9 (Ser1943) Antibody | Affinity | Cat#AF4425 | Western Blot: 1/1000 |
| Rabbit anti-USP22 | Abcam | Cat#ab195289 | Western Blot: 1/2000 |
| Rabbit IgG | Abcam | Cat# ab172730 | Immunoprecipitation: 1.5-2 μg for 1.0 mg of total protein lysate |
| MYH9 Polyclonal antibody | Proteintech | Cat#11128-1-AP | Immunoprecipitation: 1.5-2 μg for 1.0 mg of total protein lysate |
| HIF1α antibody | Proteintech | Cat# 20960-1-AP | Immunohistochemistry: 1/200  Immunoprecipitation: 1.5-2 μg for 1.0 mg of total protein lysate |
| Anti-non-muscle Myosin IIA | Abcam | Cat# ab138498 | Immunofluorescence: 1/250 - 1/500. |
| HIF-1 alpha Monoclonal antibody | Proteintech | Cat# 66730-1-Ig | Immunofluorescence: 1/250 - 1/500. |
| non-muscle Myosin IIA [p Ser1943] Antibody | NOVUS | Cat# NBP3-13579 | Immunohistochemistry: 1/200 |
| Alexa Fluor® 594 AffiniPure Goat Anti-Rabbit IgG (H+L) | Jackson Immunoresearch | Cat#111-585-003 | Immunofluorescence: 1/100 - 1/800. |
| Fluorescein (FITC) AffiniPure Goat Anti-Mouse IgG (H+L) | Jackson Immunoresearch | Cat#115-095-003 | Immunofluorescence: 1/50 - 1/200. |
| GPC3 Polyclonal antibody | Proteintech | Cat# 30021-1-AP | Immunohistochemistry: 1/50 - 1/500 |
| Ki-67 (8D5) Mouse mAb | Cell Signaling Technology | Cat# 9449 | Immunohistochemistry: 1/800 - 1/3200 |

**Supplementary Table 5. Scoring method of positive rate and staining intensity**

| Score | Percentage of positive cells |
| --- | --- |
| 1 | ≤25% |
| 2 | 26%-50% |
| 3 | 51%-75% |
| 4 | ＞75% |

| Score | Staining intensity |
| --- | --- |
| 0 | Negative |
| 1 | Weak positive |
| 2 | Moderate positive |
| 3 | Strong positive |

**Supplementary Table 6. Univariate and multivariate overall survival analysis of clinicopathological variables of hepatocellular carcinoma patient**
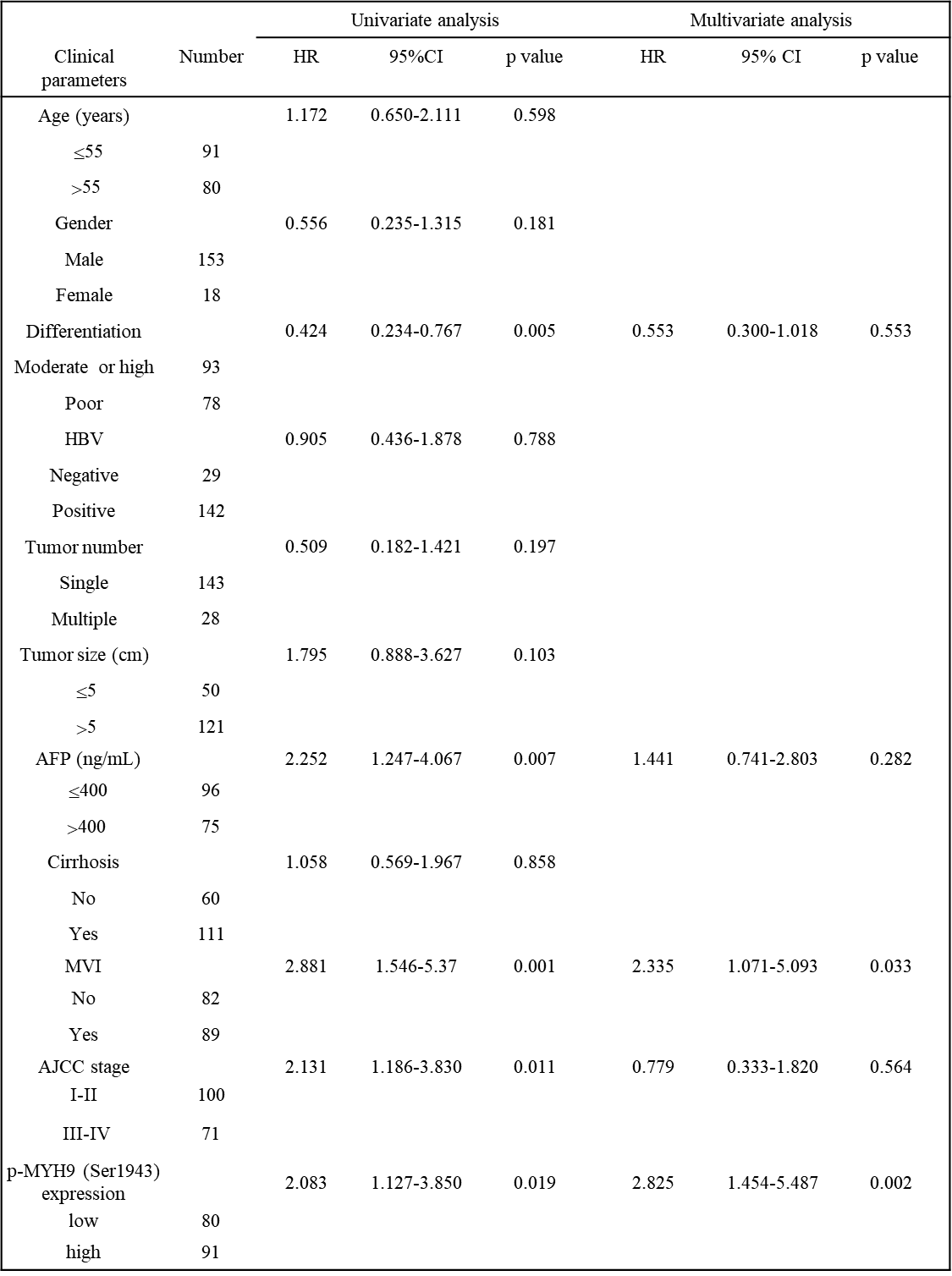


**Supplementary Table 7. Univariate and multivariate progress free survival analysis of clinicopathological variables of HCC patients using lenvatinib**


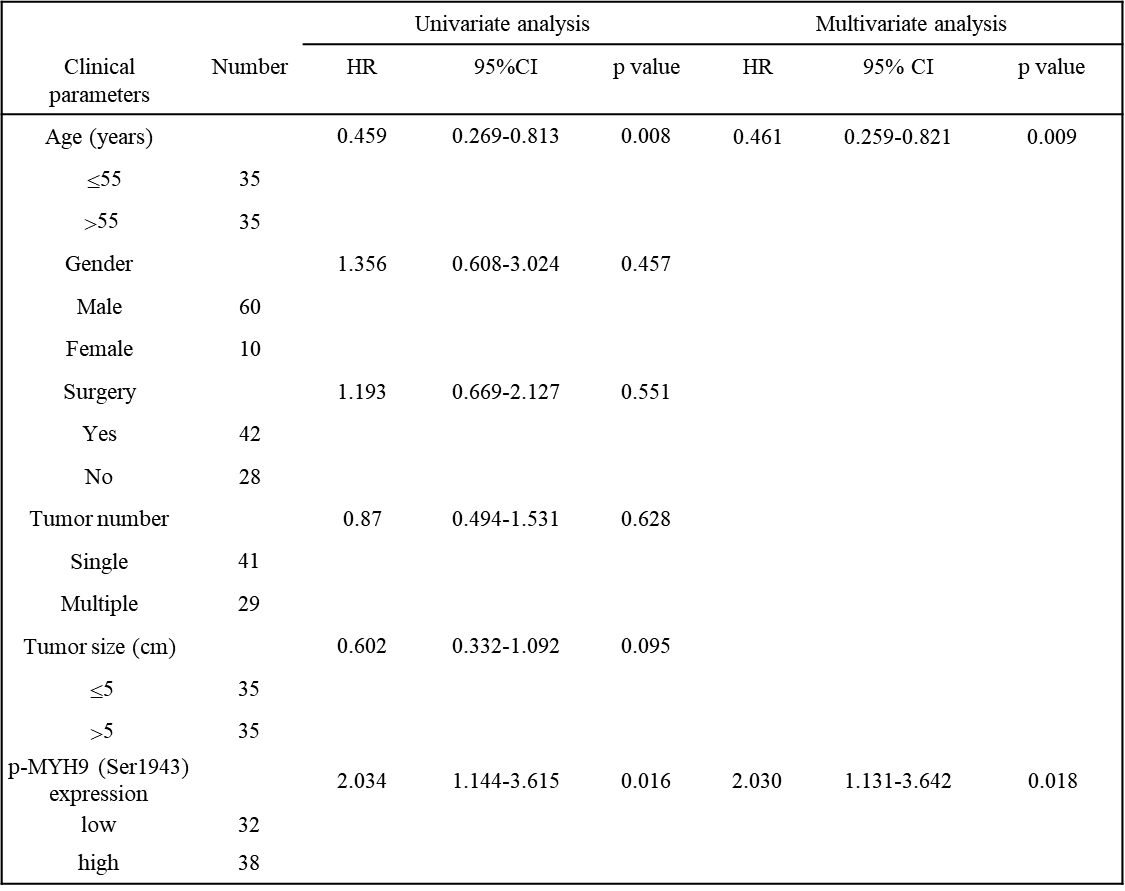


REFERENCES

1. Ling, S. *et al.* USP22 promotes hypoxia-induced hepatocellular carcinoma stemness by a HIF1α/USP22 positive feedback loop upon TP53 inactivation. *Gut* **69**, 1322-1334 (2020).
